# Social connection and mortality in UK Biobank: a prospective cohort analysis

**DOI:** 10.1186/s12916-023-03055-7

**Published:** 2023-11-10

**Authors:** Hamish M. E. Foster, Jason M. R. Gill, Frances S. Mair, Carlos A. Celis-Morales, Bhautesh D. Jani, Barbara I. Nicholl, Duncan Lee, Catherine A. O’Donnell

**Affiliations:** 1 General Practice and Primary Care, School of Health and Wellbeing, College of Medical, Veterinary and Life Sciences, University of Glasgow, Glasgow, G12 8TB Scotland; 2 School of Cardiovascular and Metabolic Health, College of Medical, Veterinary and Life Sciences, University of Glasgow, Glasgow, G12 8TA Scotland; 3 School of Mathematics and Statistics, The Mathematics and Statistics Building, University of Glasgow, Glasgow, G12 8SQ Scotland

**Keywords:** Epidemiology, Public health, Social connection, Social connection, Social isolation, Loneliness, Social determinants of health

## Abstract

**Background:**

Components of social connection are associated with mortality, but research examining their independent and combined effects in the same dataset is lacking. This study aimed to examine the independent and combined associations between functional and structural components of social connection and mortality.

**Methods:**

Analysis of 458,146 participants with full data from the UK Biobank cohort linked to mortality registers. Social connection was assessed using two functional (frequency of ability to confide in someone close and often feeling lonely) and three structural (frequency of friends/family visits, weekly group activities, and living alone) component measures. Cox proportional hazard models were used to examine the associations with all-cause and cardiovascular disease (CVD) mortality.

**Results:**

Over a median of 12.6 years (IQR 11.9–13.3) follow-up, 33,135 (7.2%) participants died, including 5112 (1.1%) CVD deaths. All social connection measures were independently associated with both outcomes. Friends/family visit frequencies < monthly were associated with a higher risk of mortality indicating a threshold effect. There were interactions between living alone and friends/family visits and between living alone and weekly group activity. For example, compared with daily friends/family visits-not living alone, there was higher all-cause mortality for daily visits-living alone (HR 1.19 [95% CI 1.12–1.26]), for never having visits-not living alone (1.33 [1.22–1.46]), and for never having visits-living alone (1.77 [1.61–1.95]). Never having friends/family visits whilst living alone potentially counteracted benefits from other components as mortality risks were highest for those reporting both never having visits and living alone regardless of weekly group activity or functional components. When all measures were combined into overall functional and structural components, there was an interaction between components: compared with participants defined as not isolated by both components, those considered isolated by both components had higher CVD mortality (HR 1.63 [1.51–1.76]) than each component alone (functional isolation 1.17 [1.06–1.29]; structural isolation 1.27 [1.18–1.36]).

**Conclusions:**

This work suggests (1) a potential threshold effect for friends/family visits, (2) that those who live alone with additional concurrent markers of structural isolation may represent a high-risk population, (3) that beneficial associations for some types of social connection might not be felt when other types of social connection are absent, and (4) considering both functional and structural components of social connection may help to identify the most isolated in society.

**Supplementary Information:**

The online version contains supplementary material available at 10.1186/s12916-023-03055-7.

## Background

Social connection is a complex phenomenon that encompasses numerous emotional, physical, and behavioural aspects of human interaction. Social connection can be classified into inter-related conceptual components, including functional (e.g. subjective feelings of loneliness) and structural components (e.g. objective frequency of social contacts) [1, 2]. Deficits of either component are associated with a higher risk of all-cause mortality and cardiovascular disease (CVD) [3–7]. The mechanisms by which components of social connection are associated with mortality are unclear and may vary by component, or by the measure used, but are thought to be mediated via direct (e.g. altered blood pressure, poorer immune function, neurodevelopmental impairment) [8–10] and indirect effects (e.g. via poorer mental health or wellbeing, lower physical activity, or higher tobacco and alcohol consumption) [11–15]. Further explanations involve reverse causality, whereby long-term health conditions or disabilities can impair people’s ability to form or sustain relationships [16, 17]. Nevertheless, the prevalence of a lack of social connection (9.2–14.4% of the global population are estimated to feel lonely and 25% of adults worldwide may be socially isolated) [18, 19] and the associated mortality justify attempts to understand how each component impacts on mortality in order to develop targeted interventions (Table 1).

**Table 1:** Components of social connection (Taken from Holt-Lundstad, 2018) [1]

**Component**	**Definition**	**Example measures**
**Functional**	Functions provided by, or perceived to be available because of, social relationships	Received support Perceptions of social support Perceived loneliness
**Structural**	Existence of and interconnections among different social relationships and roles	Marital status Living alone or not Social networks Social integration Social isolation

Often, only moderate or weak correlations are observed between different components, which may reflect the dependent and independent relationships between them [20–23]. For example, individuals with a shrinking social network might feel lonelier as a result, whilst others with a growing social network could also feel increasingly lonely if the quality of those relationships is poor. Adding to the challenge of understanding how different components of social connection are associated with adverse health outcomes is the numerous heterogeneous ways by which studies have operationalised and measured different aspects of each component [15, 24–27]. Prior studies have often focussed on a single-item measure, for example, showing that a ‘sense of loneliness’ (functional) or living alone (structural) is independently associated with a higher risk of all-cause mortality [28, 29]. Alternatively, some studies have used composite scales or indices but still with a focus on a single component of social connection (e.g. Revised UCLA Loneliness Scale measuring the functional component or the Berkman-Syme Social Network Index measuring the structural component) [30, 31]. A meta-analysis of prospective studies examining the association between both subjective (functional) or objective (structural) isolation and all-cause mortality found the average effect sizes to be similar (26–32% increased likelihood of mortality) for each type of isolation [3]. However, the effect sizes represent the aggregate effects of different measures with no consideration of the strength of the association of individual measures on health outcomes. Furthermore, meta-analyses that have quantified associations between measures of social connection and mortality have highlighted the lack of studies that include measures of both functional and structural components or that examine for potential synergistic interactions between them [3–5]. Indeed, a lack of research examining different components of social connection in the same dataset to disentangle their independent, additive, and multiplicative effects was highlighted in a recent US Surgeon General’s Advisory [32]. These are missed opportunities, as a more detailed understanding of the health impact of different components of social connection and their interactions could help guide policy and interventions designed to increase and enhance social connectedness and improve related health outcomes.

Previous studies often refer to functional and structural components of social connection as loneliness and social isolation, respectively. However, the social connection framework offers advantages for conceptualising and studying both the separate and combined effects of different social measures. Firstly, the terminology of social connection is more neutral than ‘loneliness’ or ‘isolation’ and thereby implies a spectrum of either beneficial or detrimental associations. Secondly, studies often lack methodological or theoretical underpinnings and use loneliness and social isolation as catch all phrases, which, whilst supposedly widely understood, may be interpreted differently depending on the researcher. Thirdly, social connection offers a broad framework that encapsulates both loneliness and social isolation alongside but separately from other measures of social connection. For example, a subjective feeling of loneliness can be considered a measure of the functional component but so too can perceptions of social support, which may not always be perceived as loneliness. Therefore, the terminology of the social connection framework remains flexible and inclusive whilst avoiding some assumptions around loneliness or social isolation and, as a result, could help when interpreting estimates of the separate and combined effects of various social measures.

The first aim of this study was to understand the strength of the association between independent measures of functional and structural social connection and all-cause and CVD mortality. The second aim was to understand if and how these measures interact with one another in combined associations with adverse health outcomes. Our study was guided by the following research questions (RQ):

1. What is the strength of association between two functional measures of social connection—frequency of ability to confide and perceived loneliness—and all-cause and CVD mortality, and is there an interaction between the measures?
2. What is the strength of association between three structural measures of social connection—frequency of friends and family visits, weekly leisure/social activities, and living alone—and all-cause and CVD mortality, and is there an interaction between these measures?
3. What is the pattern of the combined association between measures of (a) functional and (b) structural components of social connection and all-cause and CVD mortality?
4. Is there an interaction between functional and structural components of social connection for all-cause and CVD mortality?

## Methods

### Study design and participants

We analysed baseline data from the UK Biobank study which recruited 502,536 participants via postal invitation between 2006 and 2010. Participants attended one of 22 assessment centres in England, Scotland, or Wales to complete a questionnaire and nurse-led interview and have physical measurements taken [33]. More details of the UK Biobank procedures and assessments can be found online (biobank.ndph.ox.ac.uk/ukb/) and in the study protocol [34]. We excluded those without full data on all variables used in analyses (*n* = 44,390 [8.8%]) as detailed below (participant flowchart—Additional file 1: Fig. S1). Participants who reported ‘do not know’ or ‘prefer not to answer’ for any variable were considered missing.

### Outcome ascertainment

UK Biobank participants consented to data linkage to national mortality registers. We examined two adverse health outcomes: all-cause and CVD mortality. Any International Classification of Diseases (10th Revision) codes from I05 to I99, Z86.7, G45, and G46 given as the primary cause of death were chosen to define CVD deaths after discussion by two primary care clinicians (HMEF and FSM). These codes likely represent chronic CVD diseases, including cerebrovascular disease, with only acute rheumatic fever (I00–I02) excluded. Dates and causes of death are contained within death certificates provided by linkage to the National Health Service (NHS) Information Centre (England and Wales) and the NHS Central Register (Scotland). Censoring dates varied by country of baseline assessment (England and Wales, 30 September 2021; Scotland, 31 October 2021).

### Functional and structural component measures

Two functional and three structural component measures of social connection used in previous studies were examined in this study (Table 2) [7, 23, 35, 36]. For frequency of friend and family visits, the categories of ‘never or almost never’ and ‘no friends or family outside household’ were collapsed into a single category, ‘never’. This was justified on the basis that these responses are similar and there being few participants with no friends or family outside the household (*n* = 1031). For simplicity, categories for ordinal variables were renamed as ‘daily’, ‘2–4 times a week’, ‘weekly’, ‘monthly’, ‘once every 3 months’, and ‘never’.

**Table 2:** Functional and structural component measures and categories

**Component**	**Measure**	**Categories**
**Functional**	Frequency of ability to confide in someone close	daily, 2-4 times a week, weekly, monthly, once every 3 months, and never
	Often feeling lonely	yes, no
**Structural**	Frequency of friends and family visits	daily, 2-4 times a week, weekly, monthly, once every 3 months, and never
	Weekly group activity	yes, no
	Living alone	yes, no

### Covariate data

Baseline self-reported sex (female, male), ethnicity (White, mixed, Asian or Asian British, Black or Black British, Chinese, or other ethnic groups), smoking status (current, never/former), alcohol intake (> vs. ≤ 35 [females] and > vs. ≤ 50 [males] weekly units of alcohol—previously identified cut-offs for high risk drinking in England and UK Biobank), [37, 38] and self-reported physical activity levels (< vs. ≥ 450 MET [metabolic equivalent of task] min per week as per UK physical activity guidelines) [39, 40] were used as potential explanatory variables. A count of baseline self-reported long-term conditions confirmed at nurse-led interview was based on a list of 43 long-term conditions [41]. Month of assessment was included as a covariate as self-reported measures of social connection may vary by season [42]. Socioeconomic position was measured using the area-based measure of deprivation and Townsend index (comprising car ownership, household overcrowding, owner occupation, and unemployment) and was based on preceding census data and postcode of residence at recruitment and analysed as a continuous variable [43]. Body mass index (BMI) was calculated by trained personnel at baseline assessment and used as a continuous measure (kg/m^2^).

### Statistical analysis

We compared those participants with complete data to those with missing data using descriptive statistics. For our main analyses, we used time-to-event analysis (Cox proportional hazard models) to examine the associations between exposures and mortality outcomes for those participants with full data only. Follow-up time was calculated as the time difference between the date of assessment and either the censor date or the date of death, whichever occurred first. Additional file 1: Table S1 shows the analyses performed and the corresponding research question each analysis addresses. Measures of social connection and the covariates included in our models may be highly correlated and lead to multicollinearity and model instability [44]. Therefore, to detect potential multicollinearity, we calculated generalised variance inflation factors (GVIF) for all variables included in our Cox models using a linear regression model with follow-up time as the outcome [45].

### Functional component analyses

First, we examined the association between each functional component measure (frequency of ability to confide in someone close and often feeling lonely) and adverse health outcomes separately, adjusting for the known and likely confounders: sex, ethnicity, Townsend index, and month of assessment, smoking status, alcohol intake, physical activity, BMI, long-term condition count, frequency of friend and family visits, weekly group activity, living alone, and mutually for frequency of ability to confide/often feeling lonely (analyses 1 and 2, Additional file 1: Table S1). Next, we examined the combined association of both functional component measures (with a single reference group of almost daily ability to confide in someone close and not often feeling lonely) and their interactions for adverse health outcomes (analysis 3, Additional file 1: Table S1). To provide sufficient data for interpretation, we explored the interactions on both multiplicative and additive scales by calculating estimates for multiplicative interaction, relative excess risk of interaction (RERI), attributable portion (AP), and a synergy index (SI) [46]. The interaction tests require four exposure groups which meant dichotomising the ordinal variable of frequency of ability to confide in someone close. To inform dichotomisation, we used results from the independent and mutually adjusted association between the ordinal variable and adverse health outcomes. Therefore, the dichotomous confide variable was coded as (≥ once every 3 months vs. never). To examine the interactions between functional and structural components, we created a new dichotomous ‘functional isolation’ variable. Functional isolation was defined using the independent and mutually adjusted associations with adverse health outcomes of each functional component measure and therefore coded as either never able to confide or (yes) often feeling lonely. We examined the associations between this new variable and adverse health outcomes (analysis 4, Additional file 1: Table S1).

### Structural component analyses

Next, we examined the association between each of the structural component measures (frequency of friends and family visits, weekly group activity, and living alone) and adverse health outcomes separately, with models adjusted as above but with mutual adjustment for each structural measure and the new functional isolation variable (analyses 5–7, Additional file 1: Table S1). Then, to examine the joint associations and interactions between the structural component measures, we examined the associations and interactions between (1) frequency of friends and family visits and engagement in weekly group activity, (2) frequency of friends and family visits and living alone, and (3) weekly group activity and living alone (analyses 8–9 and 11, Additional file 1: Table S1). To examine the interactions, we dichotomised the ordinal variable of frequency of friends fand family visits as ≥ monthly/ < monthly based on its independent and mutually adjusted associations with adverse health outcomes. Where there was evidence for interaction, we also examined the stratified associations (analyses 10 and 12, Additional file 1: Table S1). We then combined the three structural component measures into a new dichotomous ‘structural isolation’ variable, coding structural isolation as having less than monthly friends and family visits or no weekly group activity or living alone. We examined the association between this new variable and adverse health outcomes (analysis 13, Additional file 1: Table S1).

### Functional and structural components together

To examine the combined effect of functional and structural measures together and to assess the impact on any dose–response relationship, we examined the associations between (1) frequency of ability to confide in someone close, often feeling lonely, structural isolation, and adverse health outcomes (analysis 14, Additional file 1: Table S1) and (2) frequency of friends and family visits, weekly group activity, living alone, functional isolation, and adverse health outcomes (analysis 15, Additional file 1: Table S1). Finally, we examined the combined associations and interaction between the two new overall functional and structural isolation variables and adverse health outcomes (analysis 16, Additional file 1: Table S1).

### Sensitivity analyses

Accounting for participants’ prior health status is critical for estimating the associations between social connection and adverse health [3]. To reduce the chance that findings could be explained by reverse causality (e.g. where poor baseline health status would explain both reduced social connectedness and higher mortality), we repeated all analyses after excluding all those who reported having CVD (diabetes, coronary heart disease, atrial fibrillation, chronic heart failure, chronic kidney disease, hypertension, stroke/transient ischaemic attack, or peripheral vascular disease) or cancer at baseline as well as those who died within 2 years of recruitment (analysis 17, Additional file 1:Table S1).

All analyses were conducted using the R statistical software version 4.2.0.

## Results

### Descriptive statistics

A total of 44,390 (8.8%) participants with missing data were excluded. Compared with those with complete data, participants with missing data were more likely to be male, older, from minority ethnic backgrounds, have been assessed in spring or summer months (April to September), be from more deprived areas, be current smokers, have low physical activity levels, have a higher BMI, and have more long-term conditions (Additional file 1: Table S2). After excluding those without full data, 458,146 (91.2%) UK Biobank participants were included in the main analyses. The mean age of participants was 56.5 years (standard deviation [SD] 8.1; range 38–73), 54.7% were women, and 95.5% were of white ethnicity (Table 3). Generally, compared to all participants, those reporting any measure of reduced social connection were more likely to be from a minority ethnic background, be more deprived, engage in more unhealthy behaviours (smoking, high alcohol intake, and low physical activity levels), have a higher BMI, and have more long-term conditions. Of those who reported each measure of reduced social connection, there was variation in the percentage who were female: often feeling lonely (62.9% women), not engaging in weekly group activities (55.1% women), living alone (58.5% women), never able to confide in someone close (40.9% women), and friend and family visits less than monthly (42.0% women). GVIF values, calculated to detect multicollinearity, ranged from 1.00 to 1.16 and were well below the proposed threshold of 10 (Additional file 1: Table S3) [44]. This reduced the concern of multicollinearity and strengthened the argument for including all the social connection measures as separate variables in the models.

**Table 3:** Descriptive characteristics of the study participants by measures of functional and structural components of social connection

	**Functional component measures**		**Structural component measures**			**Total**
	**Never able to confide in someone close**	**Often feels lonely**	**Friends and family visits less than monthly**	**Does not engage in weekly group activities**	**Lives alone**	
***N***	66,638	83,915	37,580	137,801	84,472	458,146
**Female**	27,285 (40.9%)	52,818 (62.9%)	15,775 (42.0%)	75,912 (55.1%)	49,409 (58.5%)	250,761 (54.7%)
**Age**	57.3 (7.9)	55.5 (8.0)	55.4 (7.9)	56.0 (8.0)	57.8 (7.9)	56.5 (8.1)
**Ethnicity**						
White	63,067 (94.6%)	78,476 (93.5%)	34,328 (91.3%)	131,333 (95.3%)	80,670 (95.5%)	437,462 (95.5%)
Mixed	424 (0.6%)	653 (0.8%)	305 (0.8%)	890 (0.6%)	622 (0.7%)	2646 (0.6%)
Asian^a^	1166 (1.7%)	1866 (2.2%)	872 (2.3%)	2412 (1.8%)	717 (0.8%)	6931 (1.5%)
Black^b^	1181 (1.8%)	1748 (2.1%)	1198 (3.2%)	1625 (1.2%)	1615 (1.9%)	6499 (1.4%)
Chinese	233 (0.3%)	202 (0.2%)	248 (0.7%)	452 (0.3%)	161 (0.2%)	1148 (0.3%)
‘Others’	567 (0.9%)	970 (1.2%)	629 (1.7%)	1089 (0.8%)	687 (0.8%)	3460 (0.8%)
**Month of assessment**						
January	4831 (7.2%)	5693 (6.8%)	2600 (6.9%)	9858 (7.2%)	5834 (6.9%)	32,468 (7.1%)
February	5655 (8.5%)	6880 (8.2%)	3009 (8.0%)	11,285 (8.2%)	7086 (8.4%)	37,992 (8.3%)
March	6638 (10.0%)	8329 (9.9%)	3657 (9.7%)	13,711 (9.9%)	8520 (10.1%)	45,314 (9.9%)
April	5842 (8.8%)	7305 (8.7%)	2986 (7.9%)	11,843 (8.6%)	7189 (8.5%)	39,690 (8.7%)
May	6792 (10.2%)	8879 (10.6%)	3826 (10.2%)	14,199 (10.3%)	8886 (10.5%)	46,858 (10.2%)
June	6697 (10.0%)	8752 (10.4%)	3850 (10.2%)	14,259 (10.3%)	8951 (10.6%)	46,677 (10.2%)
July	5661 (8.5%)	7359 (8.8%)	3329 (8.9%)	12,063 (8.8%)	7230 (8.6%)	38,956 (8.5%)
August	4927 (7.4%)	6373 (7.6%)	2788 (7.4%)	10,592 (7.7%)	6479 (7.7%)	34,372 (7.5%)
September	4601 (6.9%)	5961 (7.1%)	2666 (7.1%)	9688 (7.0%)	5826 (6.9%)	32,942 (7.2%)
October	5501 (8.3%)	7016 (8.4%)	3277 (8.7%)	11,378 (8.3%)	6822 (8.1%)	38,783 (8.5%)
November	5576 (8.4%)	6861 (8.2%)	3294 (8.8%)	11,220 (8.1%)	6976 (8.3%)	38,202 (8.3%)
December	3917 (5.9%)	4507 (5.4%)	2298 (6.1%)	7705 (5.6%)	4673 (5.5%)	25,892 (5.7%)
**Winter assessment**^c^	32,118 (48.2%)	39,286 (46.8%)	18,135 (48.3%)	65,157 (47.3%)	39,911 (47.2%)	218,651 (47.7%)
**Townsend index**	− 0.99 (3.27)	− 0.63 (3.34)	− 0.77 (3.36)	− 1.14 (3.14)	− 0.03 (3.43)	− 1.39 (3.04)
**Current smoker**	9035 (13.6%)	12,673 (15.1%)	4983 (13.3%)	18,510 (13.4%)	13,083 (15.5%)	47,234 (10.3%)
**High alcohol intake**	6747 (10.1%)	7625 (9.1%)	3900 (10.4%)	9238 (6.7%)	8400 (9.9%)	41,125 (9.0%)
**Low physical activity**	15,755 (23.6%)	20,149 (24.0%)	8778 (23.4%)	37,802 (27.4%)	16,946 (20.1%)	89,942 (19.6%)
**BMI, kg/m**^**2**^	28.0 (4.96)	28.0 (5.39)	27.5 (5.05)	27.7 (5.09)	27.6 (5.20)	27.4 (4.78)
**Number of long-term conditions**	1.32 (1.31)	1.51 (1.41)	1.24 (1.27)	1.28 (1.30)	1.40 (1.35)	1.20 (1.23)

Figures given are *N* (column %) or mean (SD)

*BMI* body mass index

^a^Asian or Asian British

^b^Black or Black British

^c^October-March; higher Townsend index equates to higher levels of deprivation; high alcohol intake, > 35 (females) and > 50 (males) weekly units of alcohol; low physical activity, < 450 MET minutes per week

### Association with adverse health outcomes

After a median follow-up of 12.6 years (IQR 11.9–13.3), there were 33,135 (7.2%) deaths, of which 5112 (1.1%) were CVD deaths.

#### Functional component measures—independent associations (RQ1)

**Fig. 1:**
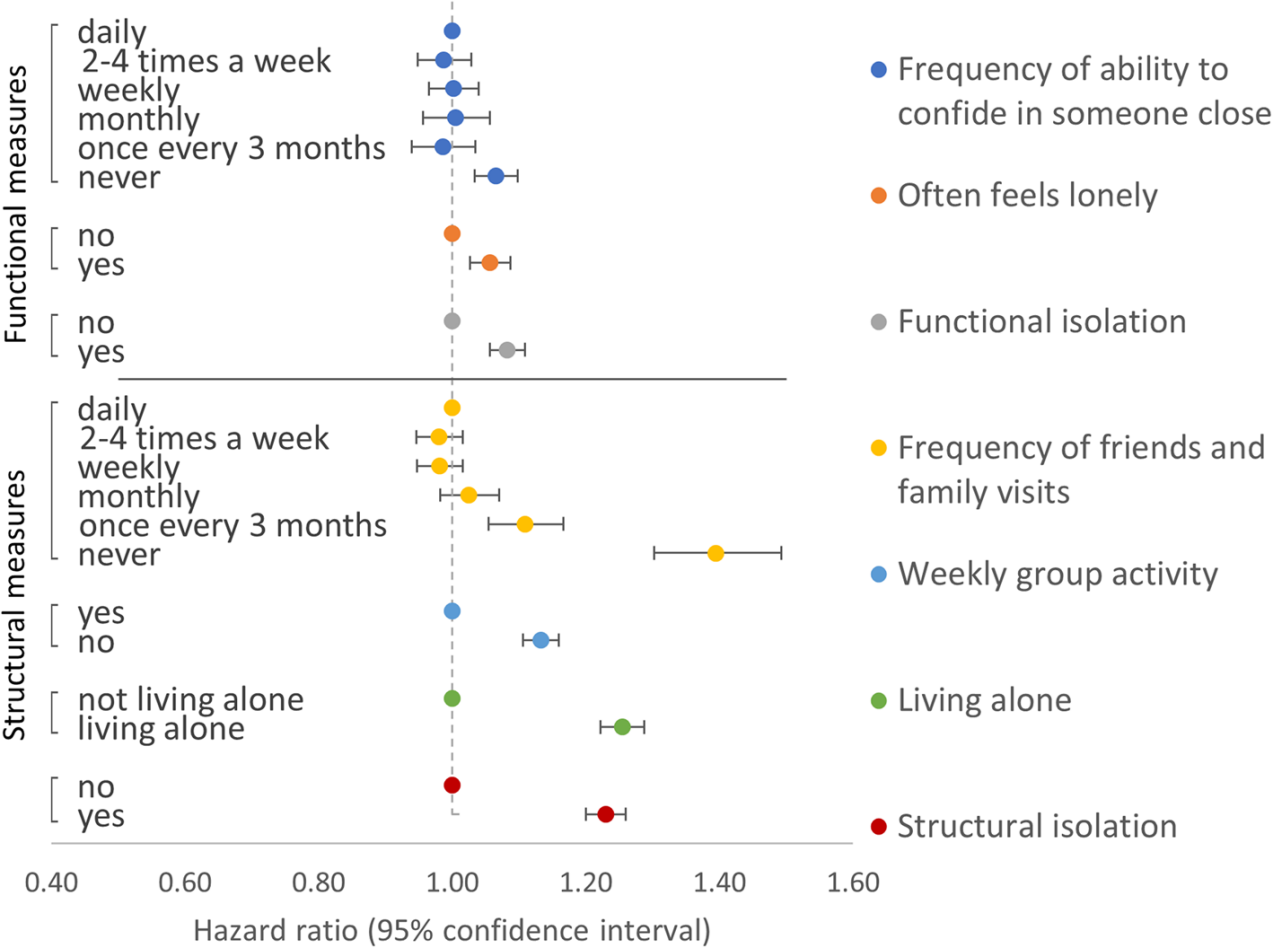
Models of association between functional and structural component measures and all-cause mortality. Models were adjusted for sex, ethnicity, Townsend index, month of assessment, smoking, alcohol, physical activity, BMI, long-term condition count, and mutually for each of the functional and structural component measures. Functional isolation defined as either never able to confide or often feels lonely. Structural isolation defined as having < monthly friends and family visits or not engaging in weekly group activity or living alone

Models of the association between the frequency of the ability to confide in someone close and outcomes showed that participants who reported never being able to confide were associated with higher all-cause and CVD mortality compared with the reference group of those who reported being able to confide daily: HR 1.07 (95% CI 1.03–1.10) and 1.17 (1.09–1.26), respectively (Table 4 and Fig. 1). Indeed, for both outcomes, there were no substantial differences in effect sizes across all categories of frequency in the ability to confide in someone close apart from never able to confide. Models of the association between often feeling lonely and outcomes showed that compared to those who reported not often feeling lonely, those often feeling lonely were also associated with higher all-cause and CVD mortality: HR 1.06 (1.03–1.09) and 1.08 (1.00–1.16) (Table 4 and Fig. 1).

**Table 4:** Models of association between functional component measures and all-cause and CVD mortality

Outcome	Measure	Number	Deaths (%)	HR	LCI	UCI
**All-cause mortality**	***Frequency of ability to confide in someone close***					
	Daily	246,851	16,588 (6.7%)	1 (ref)	**–**	**–**
	2–4 times a week	44,267	2787 (6.3%)	0.99	0.95	1.03
	Weekly	50,320	3556 (7.1%)	1.00	0.96	1.04
	Monthly	24,403	1766 (7.2%)	1.01	0.96	1.06
	Once every 3 months	25,667	1893 (7.4%)	0.99	0.94	1.03
	Never	66,638	6545 (9.8%)	1.07	1.03	1.10
	***Often feels lonely***					
	No	374,231	26,182 (7.0%)	1 (ref)	–	–
	Yes	83,915	6953 (8.3%)	1.06	1.03	1.09
	***Functional isolation***^a^					
	No	329,312	21,831 (6.6%)	1 (ref)	–	–
	Yes	128,834	11,304 (8.8%)	1.08	1.06	1.11
**CVD mortality**	***Frequency of ability to confide in someone close***					
	Daily	246,851	2425 (1.0%)	1 (ref)	–	–
	2–4 times a week	44,267	380 (0.9%)	0.96	0.86	1.07
	Weekly	50,320	504 (1.0%)	0.99	0.90	1.09
	Monthly	24,403	272 (1.1%)	1.06	0.93	1.20
	Once every 3 months	25,667	300 (1.2%)	1.05	0.93	1.18
	Never	66,638	1231 (1.8%)	1.17	1.09	1.26
	***Often feels lonely***					
	No	374,231	3932 (1.1%)	1 (ref)	–	–
	Yes	83,915	1180 (1.4%)	1.08	1.00	1.16
	***Functional isolation***^a^					
	No	329,312	3140 (1.0%)	1 (ref)	–	–
	Yes	128,834	1972 (1.5%)	1.16	1.09	1.23

Models adjusted for sex, ethnicity, Townsend index, month of assessment, smoking, alcohol, physical activity, BMI, long-term condition count, frequency of friend and family visits, weekly group activity, living alone, and mutually for frequency of ability to confide in someone close and often feels lonely

*HR* hazard ratio, *LCI* lower confidence interval, *UCI* upper confidence interval

^a^Functional isolation defined as never able to confide in someone close or often feels lonely for which models were adjusted as above but without adjusting for the frequency of ability to confide in someone close or often feels lonely

#### Functional component measures—combined associations and interactions (RQ1)

Models examining the combined associations (Additional file 1: Table S4) and interactions (Additional file 1: Table S5) between the frequency of ability to confide and often feeling lonely for adverse health outcomes did not provide clear evidence for interaction on either multiplicative or additive scales. Based on the pattern of their independent and mutually adjusted associations across both outcomes (Table 4), we combined both measures into a new dichotomous functional isolation variable, with isolation coded as reporting either never able to confide, often feeling lonely, or both. Compared to those with no functional isolation (self-reporting able to confide at least every 3 months and not often lonely), participants with functional isolation were associated with higher all-cause and CVD mortality: HR 1.08 (1.06–1.11) and 1.16 (1.09–1.23) (Table 4 and Fig. 1).

#### Structural component measures—independent associations (RQ2)

Fully adjusted models of associations between the frequency of friends and family visits and all-cause mortality showed that participants who reported visits with friends and family less often than once a month were associated with substantially higher risk of all-cause mortality: HRs (95% CI) for once every 3 months and never were 1.11 (1.05–1.17) and 1.39 (1.30–1.49), respectively (Table 5 and Fig. 1). The same pattern was observed for CVD mortality but with stronger associations and wider confidence intervals (Table 5). Compared with those who reported engaging in weekly group activity, those who reported not engaging in weekly group activity had higher all-cause and CVD mortality: HRs (95% CIs) were 1.13 (1.11–1.16) and 1.10 (1.04–1.17), respectively (Table 5 and Fig. 1). Equivalent estimates for those who reported living alone, compared with those who lived with at least one other, were 1.25 (1.22–1.29) and 1.48 (1.38–1.57) (Table 5 and Fig. 1).

**Table 5:** Models of association between structural component measures and all-cause and CVD mortality

Outcome	Measure	Number	Deaths (%)	HR	LCI	UCI
**All-cause mortality**	***Frequency of friends and family visits***					
	Daily	53,581	4548 (8.5%)	1 (ref)		
	2–4 times a week	141,881	10,491 (7.4%)	0.98	0.95	1.02
	Weekly	163,720	10,693 (6.5%)	0.98	0.95	1.02
	Monthly	61,384	4021 (6.6%)	1.02	0.98	1.07
	Once every 3 months	30,026	2327 (7.7%)	1.11	1.05	1.17
	Never	7554	1055 (14.0%)	1.39	1.30	1.49
	***Engages in weekly group activity***					
	Yes	320,345	22,047 (6.9%)	1 (ref)		
	No	137,801	11,088 (8.0%)	1.13	1.11	1.16
	***Lives alone***					
	No	373,674	24,228 (6.5%)	1 (ref)		
	Yes	84,472	8907 (10.5%)	1.25	1.22	1.29
	***Structural isolation***^a^					
	No	242,570	14,952 (6.2%)	1 (ref)	–	–
	Yes	215,576	18,183 (8.4%)	1.23	1.20	1.26
**CVD mortality**	**Frequency of friends and family visits**					
	Daily	53,581	694 (1.3%)	1 (ref)		
	2–4 times a week	141,881	1524 (1.1%)	0.95	0.86	1.04
	Weekly	163,720	1614 (1.0%)	0.95	0.87	1.04
	Monthly	61,384	627 (1.0%)	0.99	0.89	1.11
	Once every 3 months	30,026	418 (1.4%)	1.16	1.03	1.32
	Never	7554	235 (3.1%)	1.53	1.32	1.78
	**Engages in weekly group activity**					
	No	320,345	3367 (1.1%)	1 (ref)		
	Yes	137,801	1745 (1.3%)	1.10	1.04	1.17
	**Lives alone**					
	No	373,674	3547 (0.9%)	1 (ref)		
	Yes	84,472	1565 (1.9%)	1.48	1.38	1.57
	***Structural isolation***^a^					
	No	242,570	2139 (0.9%)	1 (ref)	–	–
	Yes	215,576	2973 (1.4%)	1.35	1.28	1.43

Models adjusted for sex; ethnicity; Townsend index; month of assessment; smoking; alcohol; physical activity; BMI; long-term condition count; new dichotomous loneliness variable—never or almost never able to confide in someone close OR often feeling lonely; and mutually for frequency of friend and family visits, weekly group activity, and living alone

*HR* hazard ratio, *LCI* lower confidence interval, *UCI* upper confidence interval

^a^Structural isolation defined as friends and family visits < monthly or no weekly group activity or living alone for which models were adjusted as above but without adjusting for frequency of friends and family visits, weekly group activity, or living alone

#### Structural component measures—combined associations and interactions (RQ2)

##### Frequency of friends and family visits and weekly group activity

**Fig. 2:**
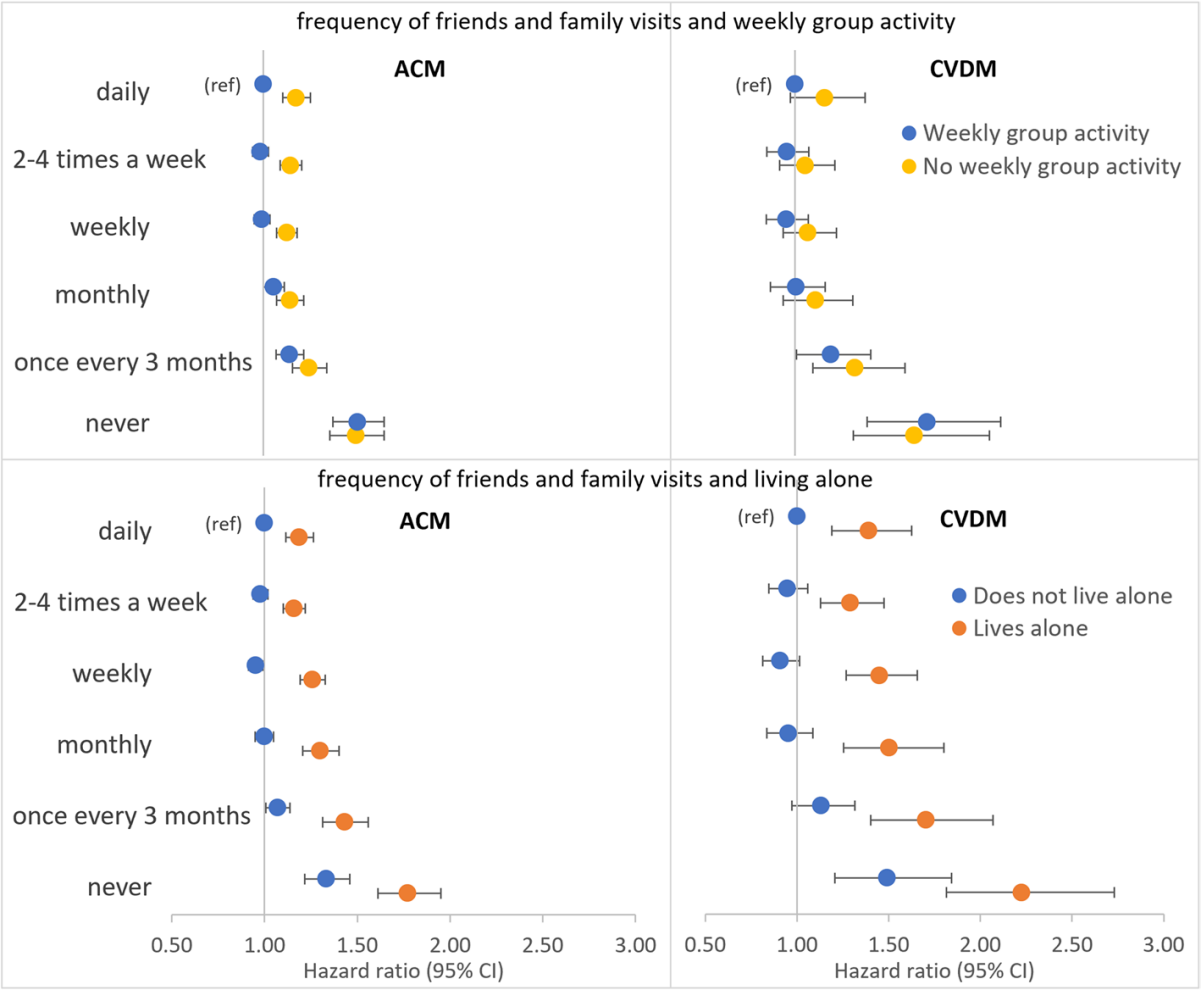
Models of combined associations between frequency of friends and family visits, weekly group activity or living alone, and all-cause (ACM) or CVD mortality (CVDM). Models adjusted for sex, ethnicity, Townsend index, month of assessment, smoking, alcohol intake, physical activity level, body mass index, long-term condition count, and mutually for weekly group activity, living alone, and functional isolation

Models of combined associations between frequency of friends and family visits and weekly group activity (reference group of daily friends and family visits and engaging in weekly group activity) showed higher all-cause mortality associated with never having friends and family visits irrespective of whether participants reported engaging in weekly group activity (HR 1.50 [1.37–1.64]) or not (HR 1.49 [1.36–1.65]) (Fig. 2 and Additional file 1: Table S6). A similar pattern was present for CVD mortality (Fig. 2 and Additional file 1: Table S6). There was a lack of evidence for an interaction between the two exposures of friend and family visit frequency (≥ monthly versus < monthly) and weekly group activity for both all-cause and CVD mortality (Additional file 1: Table S7).

##### Frequency of friends and family visits and living alone

Combined associations between frequency of friends and family visits and living alone (reference group of daily friends and family visits and not living alone) showed those who reported living alone had markedly stronger associations with each of the adverse health outcomes at every level of friend and family visit frequency (Fig. 2 and Additional file 1: Table S8). For example, compared with daily friends and family visits and not living alone, all-cause mortality HRs for those who reported never having friends and family visits were 1.33 (1.22–1.46) in those not living alone and 1.77 (1.61–1.95) in those living alone. Tests for interaction provided some evidence for a multiplicative interaction between friend and family visit frequency and living alone for all-cause (HR 1.11 [1.03–1.20]) but less so for CVD mortality (HR 1.07 [0.90–1.27]) (Additional file 1: Table S9). However, tests were suggestive of an additive interaction for CVD mortality (RERI 0.27 [− 0.01, 0.57]; AP 0.13 [− 0.02, 0.25]; SI 1.35 [0.99, 1.86]). This was consistent with the markedly higher HRs for CVD mortality in those never having friends and family visits who also lived alone (HR 2.23 [1.82–2.73]) compared with those never having friends and family visits but not living alone (HR 1.49 [1.21–1.84]) (Additional file 1: Table S8). In view of the evidence for interaction, stratified models were performed. Examining participants who reported not living alone and living alone separately showed that the relative association with all-cause mortality of never having friends and family visits, compared to daily visits, was very similar in those living alone HR (1.40 [1.26–1.55]) as when the same comparison was made in those not living alone (HRs 1.36 [1.24–1.50]) (Additional file 1: Table S10). The same pattern was seen for CVD mortality (Additional file 1: Table S10). This is consistent with a stronger independent association with adverse health outcomes for never having friends and family visits compared with living alone (Table 5 and Fig. 1).

##### Weekly group activity and living alone

Combined associations between weekly group activity and living alone (reference group of [yes] engaging in weekly group activity and not living alone) showed those who reported living alone had markedly stronger associations with each of the adverse health outcomes whether they engaged in weekly group activity or not (Additional file 1: Table S11). For example, compared with those who reported engaging in weekly group activity and not living alone, all-cause mortality HRs for those who reported no weekly group activity were 1.11 (1.08–1.14) in those not living alone and 1.46 (1.40–1.52) in those living alone. Tests for interaction provided some evidence for a multiplicative interaction between weekly group activity and living alone for all-cause (HR 1.07 [1.02–1.13]) but less so for CVD mortality (HR 1.05 [0.93–1.19]) (Additional file 1: Table S12). However, tests were more suggestive of an additive interaction for CVD mortality (RERI 0.12 [− 0.06, 0.30]; AP 0.07 [− 0.04, 0.17]; SI 1.23 [0.90, 1.67]). Models stratified by living alone showed that, compared with those who reported engaging weekly group activity, the association with all-cause mortality for those not engaging in weekly group activity was higher among those living alone (HR 1.19 [1.14–1.25]) than when the same comparison was made among those not living alone (HRs 1.11 [1.08–1.14]) (Additional file 1: Table S13). The same pattern was seen for CVD mortality (Additional file 1: Table S13).

##### Frequency of friends and family visits, weekly group activity, and living alone combined

Based on the pattern of their independent associations with both adverse health outcomes, we combined all three structural component measures into an overall dichotomous structural isolation variable, with isolation coded as < monthly friends or family visits, or not engaging in weekly group activity, or living alone. Compared to those without, participants with structural isolation were associated with higher all-cause and CVD mortality: HR 1.23 (1.20–1.26) and 1.35 (1.28–1.43) (Table 4 and Fig. 1).

#### Functional and structural component measures—combined associations and interactions

##### Frequency of ability confide, often feeling lonely, and structural isolation (RQ3)

**Fig. 3:**
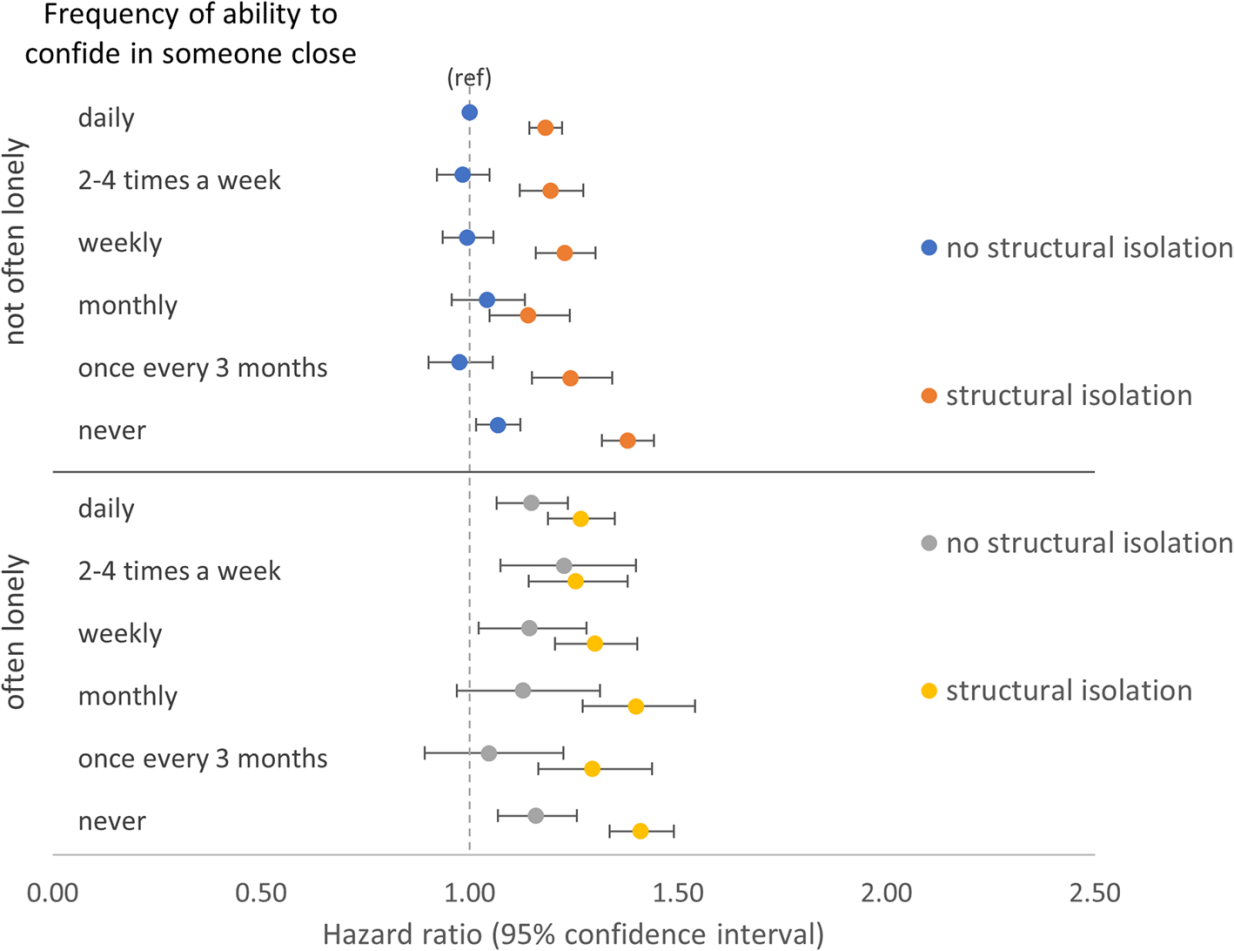
Models of combined associations between frequency of ability to confide in someone close, often feeling lonely, structural isolation, and all-cause mortality

Examining the combined associations between the two functional component measures, structural isolation, and all-cause mortality showed that, when structural isolation was present, reporting never being able to confide was associated with similarly higher all-cause mortality regardless of often feeling lonely (HR 1.41 [1.34–1.49]) or not (HR 1.38 [1.32–1.44]) (Fig. 3 and Additional file 1: Table S14). However, when structural isolation was absent, there was a greater difference in all-cause mortality associated with reporting never able to confide between those reporting often feeling lonely (HR 1.16 [1.07–1.26]) versus those reporting not often lonely (HR 1.07 [1.02–1.12]). A similar pattern was present for CVD mortality but with stronger associations and wider confidence intervals (Additional file 1: Fig. S2 and Table S14).

##### Frequency of friends and family visits, weekly group activity, living alone, and functional isolation (RQ3)

**Fig. 4:**
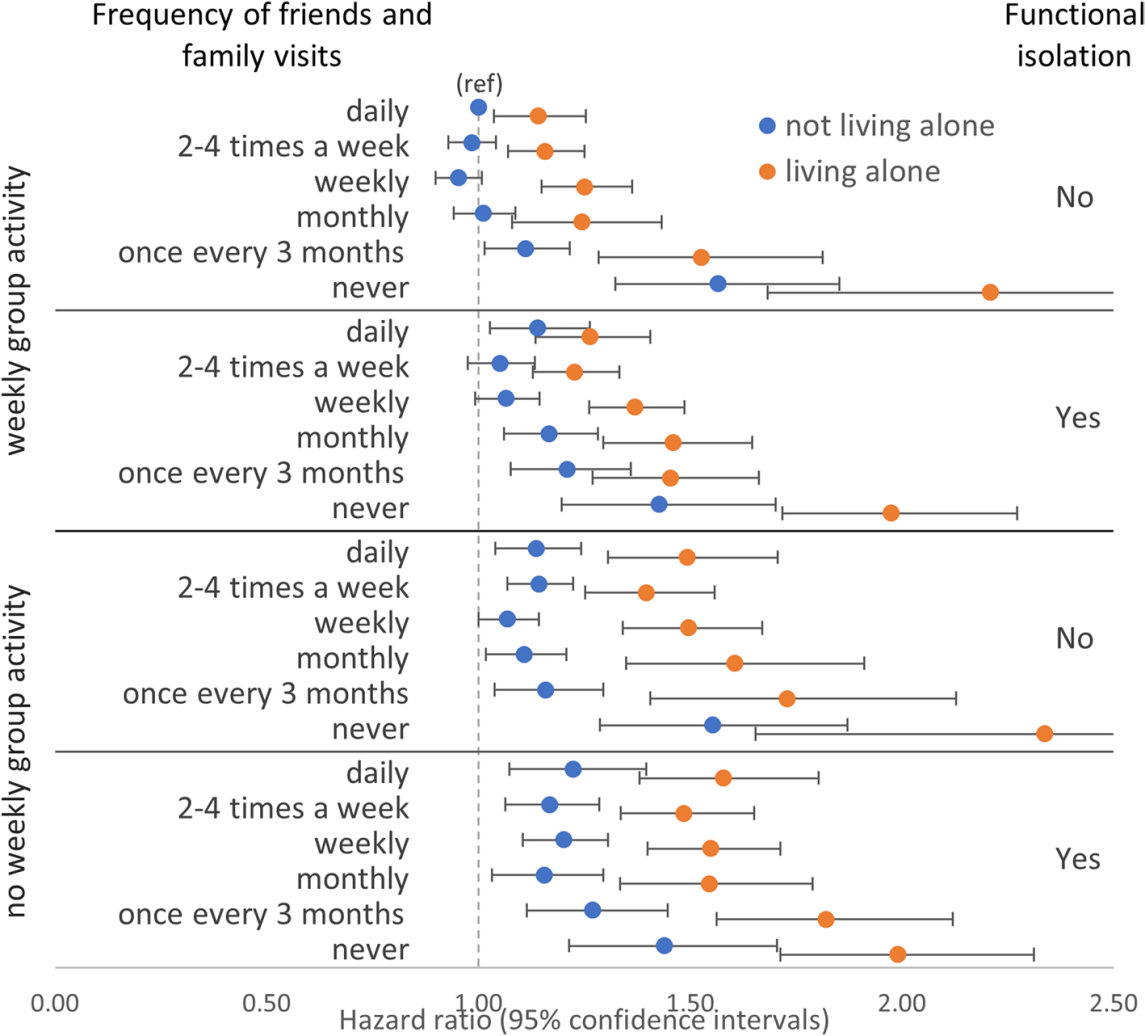
Models of combined associations between frequency of friends and family visits, weekly group activity, living alone, functional isolation, and all-cause mortality

Joint associations between all three structural component measures and functional isolation showed that, compared to the reference group of those who reported daily friends and family visits, weekly group activity, not living alone, and without functional isolation, generally, there was a dose–response relationship where the addition of any of the three structural component measures or the addition of functional isolation was associated with higher all-cause mortality (Fig. 4 and Additional file 1: Table S15). The highest all-cause mortality was observed in those who reported never having friends and family visits, not engaging weekly group activity, and living alone, but without functional isolation (HR 2.34 [1.65–3.30]). However, at this maximal level of structural isolation, there were relatively few participants without functional isolation (*n* = 170) leading to wide confidence intervals in this group and complete overlap with the estimate for otherwise equivalent participants but who did report functional isolation (HR 1.99 [1.71–2.31]). Similarly, there were comparable estimates with wide and almost completely overlapping confidence intervals for those who reported never having friends or family visits and living alone, but who also reported engaging in weekly group activity, either with functional isolation (HR 1.98 [1.72–2.27]) or without functional isolation (HR 2.21 [1.68–2.90]). A similar pattern was present when CVD mortality was modelled as the outcome but with wider confidence intervals making interpretations more challenging (Additional file 1: Fig. S3 and Table S15). Overall, this is consistent with the larger independent effects of never having friends and family visits and living alone compared with weekly group activity or functional isolation (Fig. 1).

Furthermore, in all categories of each of weekly group activity (yes/no), living alone (yes/no), and functional isolation (yes/no), there was incrementally lower all-cause mortality associated with increasing frequency in friends and family visits up to a level of monthly with further increases in frequency in friends and family visits being associated with similar levels of all-cause and CVD mortality (Fig. 4 and Additional file 1: Table S15). This is consistent with the independent effect of frequency of friends and family visits (Table 5) where visit frequencies less than monthly were associated with adverse health outcomes. This suggests there may be a threshold effect for this type of social contact above or below which the health benefits may be felt or not.

In those not living alone and with no functional isolation, not engaging in weekly group activity was associated with higher all-cause mortality compared to engaging in weekly group activity at each level of friends and family visit frequency apart from those who reported never having friends and family visits where the mortality was similar (Fig. 4). The same was true in those not living alone but with functional isolation and the pattern was more striking still in those reporting living alone.

##### Functional and structural isolation (RQ4)

**Fig. 5:**
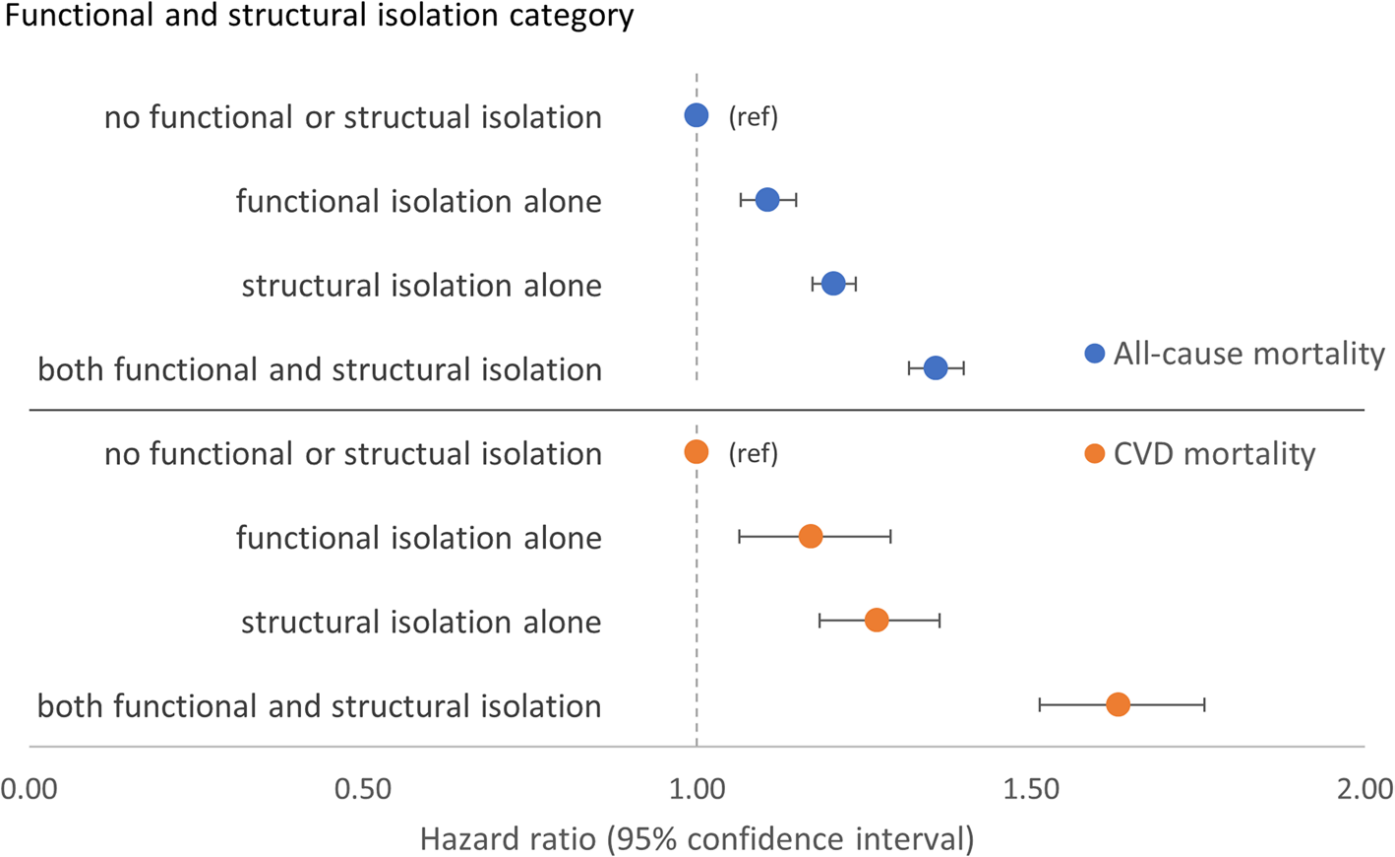
Models of combined associations between categories of functional and structural isolation and adverse health outcomes

Combined associations of functional and structural components overall showed, compared to those with neither functional nor structural isolation, there was higher all-cause mortality associated with structural isolation alone (HR 1.21 [1.17–1.24]) than with functional isolation alone (HR 1.11 [1.06–1.15]) (Fig. 5 and Additional file 1: Table S16). However, participants with both components of isolation were associated with the highest all-cause mortality (HR 1.36 [1.32–1.40]). Consistent with this were results from tests for interaction which suggested an additive interaction: RERI 0.05 [− 0.01, 0.10], AP 0.03 [− 0.01, 0.07], and SI 1.15 [0.97, 1.37] (Additional file 1: Table S17). The pattern was accentuated for CVD mortality and there was evidence of an additive interaction (Fig. 5 and Additional file 1: Tables S16 and S17).

### Sensitivity analyses

Similar results were seen across all sensitivity analyses where we excluded those with prior CVD or cancer or who died within 2 years of recruitment, albeit often with stronger associations and wider confidence intervals (Additional file 2: Tables S18-S35).

## Discussion

### Principal findings

This study shows that two measures of the functional component and three measures of the structural component of social connection were independently associated with all-cause and CVD mortality.

A combination of both measures of the functional component was also associated with adverse health outcomes. Previous studies using these measures to define ‘loneliness’ in UK Biobank may have underestimated this component of social connection as loneliness was only defined when both criteria were met [7, 35, 36].

The association between never being able to confide in someone close and both adverse health outcomes appeared to be stronger when structural isolation was present irrespective of a subjective feeling of loneliness. When structural isolation was absent, the effect of never being able to confide appeared to be stronger among those who felt lonely. This highlights the complexity present in social connection but also why it may be important to consider different measures when exploring the combined effects of functional and structural components of social connection on health outcomes.

Friends and family visit frequency of less than monthly was associated with higher all-cause and CVD mortality suggestive of a potential threshold effect, where visits up to a level of once a month could be required to maximise the benefits associated with these contacts. Furthermore, never having friends and family visits was associated with the highest all-cause and CVD mortality of any of the measures examined, but associations were markedly lower for those reporting visits once every 3 months. If causal, this could suggest large health benefits may be associated with small changes in certain measures of social connection in those with a complete lack of that type of connection. Replicating this finding in other datasets and or identifying which measures of social connection would be most beneficial to target, and the level of change which would maximise benefit could be extremely valuable.

The independent association between living alone and both all-cause and CVD mortality and the interactions with frequency of friends and family visits and with weekly group activity seen here suggests there may be high risks for living alone and even higher risks for living alone with additional levels of structural isolation (e.g. infrequent friends and family contacts or not engaging in regular group activity). Whilst it may be difficult or undesirable to change some individuals’ living circumstances, our results suggest further investigation into whether identifying those who live alone (e.g. by front-line clinicians) could be warranted [47, 48].

When three structural component measures were examined in combination with functional isolation, the risks were similarly higher for all those with no friends or family contacts who also lived alone regardless of the presence of functional isolation or whether participants engaged in group activity. This result suggests there may be a hierarchy of components of social connection for those who experience numerous types of social disconnection. For example, our study showed the lower risk of mortality associated with regular group activity appeared to be masked by a lack of friends and family visits and living alone. Exploring this concept in other datasets could highlight targets for intervention for the most isolated in society.

Overall combined associations of functional and structural isolation showed that those defined as isolated by both components had the strongest associations with adverse health outcomes and there was evidence for an additive interaction for CVD mortality. Thus, further highlighting the potential importance of considering both components together.

### Strengths and weaknesses

This study shows the added value of examining the adverse health outcomes associated with different individual measures of functional and structural components of social connection and their joint associations and interactions. A major strength of this study includes the large sample size of UK Biobank, which allowed us to examine the combination of different measures and components of social connection whilst adjusting for numerous potential confounders. The large sample size also allowed us to conduct sensitivity analyses and show that our findings are less likely to be due to reverse causality.

There are some important limitations to our study. UK Biobank has a response rate of 5.5% and is not representative of the UK general population, which means there are risks of collider or selection bias [49]. However, whilst prevalence estimates may be inaccurate, strengths of association are likely to be more generalisable [50]. There remains a possibility of unmeasured confounding despite adjusting for numerous potential confounders. We have performed numerous analyses in this study which raises the issue of multiple testing. As a result, we only draw general conclusions where patterns of results (or differences in subgroups) are consistent across analyses of both outcomes and sensitivity analyses. The measures of social connection examined are self-reported which means our results could be affected by misclassification bias, leading to under or overestimates depending on the presence of random or systematic misclassification [51]. The measures used here are also relatively crude and binary variables fail to capture severity or dose–response relationships. There are numerous alternative measures of functional and structural components of social connection not examined in this study which may be of equal or greater importance [2]. Indeed, relationship quality (e.g. marital strain) is another key component of social connection also associated with mortality [52–54]. UK Biobank, and therefore this analysis, lacks data that assesses relationship quality for the whole cohort. However, our study shows that separate measures of different components of social connection can interact, and further research could examine additional and more complex measures in similar detail.

### Comparison with the wider literature

Previous UK Biobank analyses examining both functional and structural components of social connection have used the same measures as in our analysis but have coded the item responses into scales of loneliness and social isolation, respectively [7, 35, 36]. For example, Elovainio et al. examined the association between loneliness and social isolation and mortality using the same measures as in our analysis to create social isolation and loneliness scores, but they did not examine the association between each measure that comprised the score (frequency of friends and family visits) and for the ordinal variables (frequency of ability to confide and frequency of friends and family visits) they did not estimate the level at which these measures were associated with outcomes. Furthermore, their study did not examine the interactions between measures or between loneliness and social isolation. Our findings highlight the value of examining separate measures of functional and structural components of social connection.

Previous studies have examined the interaction between functional and structural components of social connection, but their results are mixed and are based on different multi-item scales or indices of each component making comparisons difficult. Some studies found no interaction between the two components, [7, 31, 55, 56] whilst one found a positive interaction (where higher functional isolation strengthens the association between structural isolation and mortality and vice versa) [57] and another found a negative interaction (where higher functional isolation weakens the association between structural isolation and mortality and vice versa) [58]. However, none of these studies examined additive interactions, and none examined the associations or interaction between the separate measures that make up the multi-item scales or indices. Our study shows how examining the underlying associations of separate measures that make up each component may be warranted prior to defining isolation for each component. In our study, there was evidence of an additive interaction between functional and structural components for CVD mortality and suggestive of the same for all-cause mortality. Overall, our findings highlight why considering both components together may be important, particularly when developing methods for identifying high-risk target populations for intervention.

Our findings differ from those of a meta-analysis of prospective studies examining the association between objective social isolation (e.g. infrequent social contacts), living alone, subjective loneliness, and all-cause mortality [3]. In that study, the average effect sizes were similar for social isolation, loneliness, and living alone (29%, 26%, and 32% increased likelihood of mortality, respectively). In contrast, we found greater effect sizes for those with the least frequent friends and family visits and for those who live alone compared with the effect sizes for not engaging in weekly group activity or those who felt lonely. The importance of having some friends and family visits highlighted here suggests that these contacts could represent a more valuable type of social connection than others (e.g. social contact at a weekly group). For example, these contacts could reflect high-quality social connections, and therefore, a lack of which would be strongly associated with adverse health outcomes. Additionally, these types of contacts may provide more practical support or be more likely to identify subtle deteriorations in the health and well-being of an individual. This is consistent with the smaller effect sizes for weekly group activity in our study, which featured in studies in the meta-analysis but often as part of multi-item measures of structural isolation where its individual impact was not assessed. The relatively lower effect size for functional isolation seen here compared with the equivalent results for loneliness from the meta-analysis, could be explained by a less stringent measure of functional isolation used here albeit with our measure being based on the associations between the individual constituent measures and adverse health outcomes.

Previous work has highlighted a lack of evidence for a threshold effect of measures of social connection, where risk becomes more pronounced at a certain level of isolation [3]. However, our study suggests that a threshold effect may exist as mortality associated with friends and family visits frequency was only higher at ‘about once a month’ and less often, although this result may be due to the categories available (the response items in the original questionnaire) and there may indeed be a continuum of risk.

### Future research

There is no standard measure for social connection. However, the independent risks of living alone, and the interactions with both friends and family visits and weekly group activity, seen here suggests that further work is warranted in ascertaining whether living alone could represent a single and simple measure that could be standardised and included in studies examining social connection [59]. Our findings suggest that the benefits of group activity could be masked by an overriding negative effect of never having friends or family contacts. Further examination into the ways in which components of social connection interact could inform how intervention targets might be prioritised, particularly for those who are most isolated. Finally, more work is required to understand the role of potential mediators (e.g. mental health problems or health behaviours) to further elucidate the mechanistic pathways by which social disconnection might cause adverse health outcomes and inform future interventions.

## Conclusions

This study of UK Biobank is the first to examine two measures of the functional component and three measures of the structural component of social connection both independently and in combination. Our findings suggest that advice, interventions, and policy may need to be tailored to address different aspects of social connection and target the highest risk groups. Specifically, we show that separate measures of different components of social connection may contribute different levels of risk of adverse health outcomes, and the combined associations and interactions of the measures examined here suggest that those who live alone with additional concurrent markers of structural isolation may represent a population who could benefit from targeted support.

### Supplementary Information

**Additional file 1:** **Fig. S1.** Flowchart of study participants. **Table S1.** Table of analyses. **Table S2.** Characteristics of participants with missing and complete data. **Table S3.** Generalised variance inflation factors for all variables included in Cox models. **Table S4.** Combined associations between frequency of ability to confide in someone close, feeling lonely, and adverse health outcomes. **Table S5.** Interaction estimates for adverse health outcomes for binary exposures of frequency of ability to confide in someone close and often feels lonely. **Table S6.** Combined associations between frequency of friends and family visits, engaging in weekly group activity, and adverse health outcomes. **Table S7.** Interaction estimates for adverse health outcomes for binary exposures of frequency of friends and family visits and weekly group activity. **Table S8.** Combined associations between frequency of friends and family visits, living alone, and adverse health outcomes. **Table S9.** Interaction estimates for adverse health outcomes for binary exposures of frequency of friends and family visits and living alone. **Table S10.** Associations between frequency of friends and family visits and adverse health outcomes stratified by living alone. **Table S11.** Combined associations between weekly group activity, living alone, and adverse health outcomes. **Table S12.** Interaction estimates for adverse health outcomes for binary exposures of weekly group activity and living alone. **Table S13.** Associations between weekly group activity and adverse health outcomes stratified by living alone. **Table S14.** Combined associations between frequency of ability to confide in someone close, often feeling lonely, and structural isolation, and adverse health outcomes. **Fig. S2.** Combined associations between frequency of ability to confide in someone close, often feeling lonely, structural isolation, and CVD mortality. **Table S15.** Combined associations between frequency of friends and family visits, weekly group activity, living alone, functional isolation, and adverse health outcomes. **Fig. S3.** Combined associations between frequency of friends and family visits, weekly group activity, living alone, functional isolation, and CVD mortality. **Table S16.** Associations between functional and structural isolation and adverse health outcomes. **Table S17.** Interaction estimates for adverse health outcomes for binary exposures of functional and structural isolation.

**Additional file 2:** Tables S18-S35 show results from sensitivity analyses where those with self-reported prior CVD or cancer or who died with 2 years of recruitment were excluded. **Table S18.** Associations between frequency of ability to confide in someone close and adverse health outcomes. **Table S19.** Associations between often feeling lonely and adverse health outcomes. **Table S20.** Combined associations between frequency of ability to confide in someone close, often feeling lonely and adverse health outcomes. **Table S21.** Interaction estimates for adverse health outcomes for binary exposures of never able to confide in someone close and often feeling lonely. **Table S22.** Associations between functional isolation and all-cause and CVD mortality. **Table S23.** Associations between structural component measures and all-cause and CVD mortality. **Table S24.** Combined associations between frequency of friends and family visits, engaging in weekly group activity, and adverse health outcomes. **Table S25.** Interaction estimates for adverse health outcomes for binary exposures of frequency of friends and family visits and weekly group activity. **Table S26.** Combined associations between frequency of friends and family visits, living alone, and adverse health outcomes. **Table S27.** Interaction estimates for adverse health outcomes for binary exposures of friends and family visits less than monthly and living alone. **Table S28.** Associations between frequency of friends and family visits and adverse health outcomes stratified by living alone. **Table S29.** Combined associations between weekly group activity, living alone, and adverse health outcomes. **Table S30.** Interaction estimates for adverse health outcomes for binary exposures of weekly group activity and living alone. **Table S31.** Associations between weekly group activity and adverse health outcomes stratified by living alone. **Table S32.** Combined associations between frequency of ability to confide in someone close, often feeling lonely, and structural isolation, and adverse health outcomes. **Table S33.** Combined associations between frequency of friends and family visits, weekly group activity, living alone, functional isolation, and adverse health outcomes. **Table S34.** Combined associations between functional and structural isolation and adverse health outcomes. **Table S35.** Interaction estimates for adverse health outcomes for binary exposures of functional and structural isolation.

## Data Availability

UK Biobank data is available to all bona fide researchers to perform health-related research that is in the public interest. Data access is subject to an application process. Details are available online at https://www.ukbiobank.ac.uk/.

## Abbreviations

AP: Attributable portion
BMI: Body mass index
CI: Confidence intervals
CVD: Cardiovascular disease
GVIF: Generalised variance inflation factors
IQR: Interquartile range
LCI: Lower confidence interval
MET: Metabolic equivalent of task
NHS: National Health Service
RERI: Relative excess risk of interaction
RQ: Research question
SD: Standard deviation
SI: Synergy index
UCI: Upper confidence interval
