# Supplementary material for: Social connection and mortality in UK Biobank: a prospective cohort analysis
Source: BMC Med. 2023 Nov 10;21:384. doi: 10.1186/s12916-023-03055-7 (PMC10637015; doi:10.1186/s12916-023-03055-7)
Supplement: Supplementary file 1 — Additional file 1: Fig. S1. Flowchart of study participants. Table S1. Table of analyses. Table S2. Characteristics of participants with missing and complete data. Table S3. Generalised variance inflation factors for all variables included in Cox models. Table S4. Combined associations between frequency of ability to confide in someone close, feeling lonely, and adverse health outcomes. Table S5. Interaction estimates for adverse health outcomes for binary exposures of frequency of ability to confide in someone close and often feels lonely. Table S6. Combined associations between frequency of friends and family visits, engaging in weekly group activity, and adverse health outcomes. Table S7. Interaction estimates for adverse health outcomes for binary exposures of frequency of friends and family visits and weekly group activity. Table S8. Combined associations between frequency of friends and family visits, living alone, and adverse health outcomes. Table S9. Interaction estimates for adverse health outcomes for binary exposures of frequency of friends and family visits and living alone. Table S10. Associations between frequency of friends and family visits and adverse health outcomes stratified by living alone. Table S11. Combined associations between weekly group activity, living alone, and adverse health outcomes. Table S12. Interaction estimates for adverse health outcomes for binary exposures of weekly group activity and living alone. Table S13. Associations between weekly group activity and adverse health outcomes stratified by living alone. Table S14. Combined associations between frequency of ability to confide in someone close, often feeling lonely, and structural isolation, and adverse health outcomes. Fig. S2. Combined associations between frequency of ability to confide in someone close, often feeling lonely, structural isolation, and CVD mortality. Table S15. Combined associations between frequency of friends and family visits, weekly group activity, li [file 12916_2023_3055_MOESM1_ESM.docx]

# Additional File 1

## S1 Figure

Flowchart of study participants (% total UK Biobank)

UK Biobank total

N=502,536

Exclude those with missing data

N=44,390 (8.8)

N = 502,536

**Participants included in main analyses**

**N=458,146 (91.2)**

N = 502,536

Exclude those who reported CVD at baseline

N=141,306 (28.1)

N = 502,536

Exclude those who reported cancer at baseline

N=23,676 (4.7)

N = 502,536

Exclude those who died with 2 years of recruitment

N=648 (0.1)

N = 502,536

Participants remaining

N=316,840 (63.0)

Participants remaining

N=293,164 (58.3)

**Participants included in sensitivity analyses**

**N=292,516 (58.2)**

S1 Table

Table of analyses

|  | **Analysis of association between adverse health outcomes and:** | **Corresponding research question** |
| --- | --- | --- |
| **1** | Frequency of ability to confide in someone close | RQ1 |
| **2** | Often feeling lonely | RQ1 |
| **3** | Frequency of ability to confide in someone close and often feeling lonely combined (and their interaction) | RQ1 |
| **4** | Functional isolation (never able to confide OR often feeling lonely) | RQ1 |
| **5** | Frequency of friends and family visits | RQ2 |
| **6** | Weekly group activity | RQ2 |
| **7** | Living alone | RQ2 |
| **8** | Frequency of friends and family visits and weekly group activity combined (and their interaction) | RQ2 |
| **9** | Frequency of friends and family visits and living alone combined (and their interaction) | RQ2 |
| **10** | Frequency of friends and family visits stratified by living alone | RQ2 |
| **11** | Weekly group activity and living alone combined (and their interaction) | RQ2 |
| **12** | Weekly group activity stratified by living alone | RQ2 |
| **13** | Structural isolation (friends and family visits <monthly OR no weekly group activity OR living alone) | RQ2 |
| **14** | Frequency of ability confide, often feeling lonely, and structural isolation combined | RQ3 |
| **15** | Frequency of friends and family visits, weekly group activity, living alone, and functional isolation combined | RQ3 |
| **16** | Functional and structural isolation (and their interaction) | RQ4 |
| **17** | Sensitivity analyses: analyses 1-16 repeated with participants with baseline self-reported CVD or cancer and those who died within two years of recruitment excluded | RQ1-4 |

## S2 Table

Comparison of characteristics of participants with missing and complete data.

|  | **Missing** | **Complete data** |
| --- | --- | --- |
| **N** | 44,390 | 458,146 |
| **Female** | 22,641 (51.0%) | 250,761 (54.7%) |
| **Mean age (SD)** | 56.8 (8.27) | 56.5 (8.08) |
| **Ethnicity** |  |  |
| White | 35,263 (79.4%) | 437,462 (95.5%) |
| Mixed | 312 (0.7%) | 2,646 (0.6%) |
| Asian or Asian British | 2,951 (6.6%) | 6,931 (1.5%) |
| Black or Black British | 1,562 (3.5%) | 6,499 (1.4%) |
| Chinese | 426 (1.0%) | 1,148 (0.3%) |
| Other ethnic group | 1,099 (2.5%) | 3,460 (0.8%) |
| Missing | 2,777 (6.3%) |  |
| **Month of assessment** |  |  |
| Jan | 2,875 (6.5%) | 32,468 (7.1%) |
| Feb | 3,185 (7.2%) | 37,992 (8.3%) |
| Mar | 4,298 (9.7%) | 45,314 (9.9%) |
| Apr | 4,428 (10.0%) | 39,690 (8.7%) |
| May | 5,895 (13.3%) | 46,858 (10.2%) |
| Jun | 5,328 (12.0%) | 46,677 (10.2%) |
| Jul | 3,578 (8.1%) | 38,956 (8.5%) |
| Aug | 3,088 (7.0%) | 34,372 (7.5%) |
| Sep | 2,700 (6.1%) | 32,942 (7.2%) |
| Oct | 3,348 (7.5%) | 38,783 (8.5%) |
| Nov | 3,416 (7.7%) | 38,202 (8.3%) |
| Dec | 2,251 (5.1%) | 25,892 (5.7%) |
| **Winter assessment** | 9,015 (20.3%) | 218,651 (47.7%) |
| **Townsend index** |  |  |
| Mean (SD) | -0.30 (3.49) | -1.39 (3.04) |
| Missing | 623 (1.4%) |  |
| **Smoking** |  |  |
| Current | 5,745 (12.9%) | 47,234 (10.3%) |
| Missing | 2,950 (6.6%) |  |
| **Alcohol intake** |  |  |
| High | 2,988 (6.7%) | 41,125 (9.0%) |
| Missing | 1,502 (3.4%) |  |
| **Physical activity level** |  |  |
| Low | 15,082 (34.0%) | 89,942 (19.6%) |
| Missing | 5,041 (11.4%) |  |
| **BMI, kg/m^2^** |  |  |
| Mean (SD) | 27.8 (5.04) | 27.4 (4.78) |
| Missing | 3,105 (7.0%) |  |
| **Mean number of long-term conditions (SD)** | 1.28 (1.33) | 1.20 (1.23) |

Figures given are N (column %) unless stated otherwise. Higher Townsend index equates to higher levels of deprivation. High alcohol intake, > 35 (females) and > 50 (males) weekly units of alcohol. Low physical activity, <450 MET minutes per week. BMI, body mass index.

## S3 Table

Generalised variance inflation factors (GVIF) for all variables included in Cox models

| **Variable** | **GVIF** | **Df** | **Adjusted GVIF** |
| --- | --- | --- | --- |
| Sex | 1.08 | 1 | 1.04 |
| Ethnicity | 1.09 | 19 | 1.00 |
| Month of assessment | 1.01 | 11 | 1.00 |
| Townsend index | 1.14 | 1 | 1.07 |
| Smoking status | 1.05 | 1 | 1.03 |
| Alcohol intake | 1.03 | 1 | 1.01 |
| Physical activity level | 1.04 | 1 | 1.02 |
| Body mass index | 1.10 | 1 | 1.05 |
| Number of long-term conditions | 1.09 | 1 | 1.04 |
| Frequency of ability to confide in someone close | 1.16 | 5 | 1.01 |
| Often feeling lonely | 1.13 | 1 | 1.06 |
| Friends and family visit frequency | 1.10 | 5 | 1.01 |
| Weekly group activity | 1.04 | 1 | 1.02 |
| Living alone | 1.13 | 1 | 1.06 |

GVIF, generalised variance inflation factors. Df, degrees of freedom. Adjusted GVIF, adjusted for degrees of freedom via the formula: GVIF^(1/(2*Df))

## S4 Table

Fully adjusted models of the combined association between frequency of ability to confide in someone close, feeling lonely and adverse health outcomes.

| Outcome | Often feels lonely | Frequency of ability to confide in someone close | n | Deaths (%) | HR | lci | uci |
| --- | --- | --- | --- | --- | --- | --- | --- |
| All-cause mortality | No | Daily | 221,955 | 14,715 (6.6%) | 1 (ref) | - | - |
|  |  | 2-4 times a week | 34,788 | 2,097 (6.0%) | 0.98 | 0.94 | 1.03 |
|  |  | Weekly | 36,762 | 2,492 (6.8%) | 1.00 | 0.96 | 1.04 |
|  |  | Monthly | 17,009 | 1,159 (6.8%) | 0.98 | 0.93 | 1.04 |
|  |  | Once every 3 months | 18,798 | 1,368 (7.3%) | 0.99 | 0.94 | 1.05 |
|  |  | Never | 44,919 | 4,351 (9.7%) | 1.08 | 1.04 | 1.12 |
|  | Yes | Daily | 24,896 | 1,873 (7.5%) | 1.07 | 1.02 | 1.12 |
|  |  | 2-4 times a week | 9,479 | 690 (7.3%) | 1.06 | 0.99 | 1.15 |
|  |  | Weekly | 13,558 | 1,064 (7.8%) | 1.07 | 1.00 | 1.14 |
|  |  | Monthly | 7,394 | 607 (8.2%) | 1.11 | 1.03 | 1.21 |
|  |  | Once every 3 months | 6,869 | 525 (7.6%) | 1.03 | 0.94 | 1.12 |
|  |  | Never | 21,719 | 2,194 (10.1%) | 1.10 | 1.05 | 1.15 |
|  |  |  |  |  |  |  |  |
| CVD mortality | No | Daily | 221,955 | 2,135 (1.0%) | 1 (ref) | - | - |
|  |  | 2-4 times a week | 34,788 | 284 (0.8%) | 0.97 | 0.86 | 1.10 |
|  |  | Weekly | 36,762 | 345 (0.9%) | 1.00 | 0.89 | 1.12 |
|  |  | Monthly | 17,009 | 165 (1.0%) | 0.98 | 0.84 | 1.15 |
|  |  | Once every 3 months | 18,798 | 211 (1.1%) | 1.05 | 0.91 | 1.21 |
|  |  | Never | 44,919 | 792 (1.8%) | 1.19 | 1.09 | 1.29 |
|  | Yes | Daily | 24,896 | 290 (1.2%) | 1.09 | 0.96 | 1.23 |
|  |  | 2-4 times a week | 9,479 | 96 (1.0%) | 1.00 | 0.81 | 1.23 |
|  |  | Weekly | 13,558 | 159 (1.2%) | 1.06 | 0.89 | 1.25 |
|  |  | Monthly | 7,394 | 107 (1.4%) | 1.31 | 1.07 | 1.59 |
|  |  | Once every 3 months | 6,869 | 89 (1.3%) | 1.12 | 0.91 | 1.39 |
|  |  | Never | 21,719 | 439 (2.0%) | 1.23 | 1.10 | 1.37 |

Models adjusted for sex, ethnicity, Townsend, month of assessment, smoking, alcohol, physical activity, BMI, long-term condition count, social isolation measures [frequency of friend and family visits, weekly group activity, living alone]. HR, hazard ratio; lci, lower confidence interval; uci, upper confidence interval.

S5 Table

Interaction estimates for adverse health outcomes for binary exposures of frequency of ability to confide in someone close (≥Once every 3 months vs never) and often feels lonely (no vs yes).

|  | **Interaction** | **Estimate** |
| --- | --- | --- |
| All-cause mortality | Multiplicative scale | 0.96 [0.90, 1.02] |
|  | RERI | -0.04 [-0.11, 0.03] |
|  | AP | -0.04 [-0.10, 0.02] |
|  | SI | 0.77 [0.50, 1.18] |
|  | | |
| CVD mortality | Multiplicative scale | 0.94 [0.82, 1.08] |
|  | RERI | -0.06 [-0.23, 0.11] |
|  | AP | -0.05 [-0.20, 0.08] |
|  | SI | 0.80 [0.42, 1.52] |

Models adjusted for sex, ethnicity, Townsend, month of assessment, smoking, alcohol, physical activity, BMI, long-term condition count, frequency of friend and family visits, weekly group activity, living alone. Estimates given as Hazard ratios [95% confidence intervals]).

RERI, relative excess risk for interaction; AP, attributable portion, SI, synergy index. A RERI or AP of zero means no interaction or perfect additivity. A RERI or AP of greater than zero means positive interaction or more than additivity. A RERI or AP of less than zero means negative interaction or less than additivity. An SI of greater than one means positive interaction or more than additivity. An SI of less than one means negative interaction or less than additivity.

S6 Table

Fully adjusted models of joint association between frequency of friends and family visits, engaging in weekly group activity, and adverse health outcomes.

| **Outcome** | **Weekly group activity** | **Frequency of friends and family visits** | **N** | **Deaths (%)** | **HR** | **lci** | **uci** |
| --- | --- | --- | --- | --- | --- | --- | --- |
| **All-cause mortality** | **Yes** | Daily | 38,297 | 3,090 (8.1%) | 1 (ref) | - | - |
|  |  | 2-4 times a week | 104,838 | 7,359 (7.0%) | 0.98 | 0.94 | 1.03 |
|  |  | Weekly | 114,374 | 7,115 (6.2%) | 0.99 | 0.95 | 1.03 |
|  |  | Monthly | 40,183 | 2,532 (6.3%) | 1.05 | 1.00 | 1.11 |
|  |  | Once every 3 months | 18,646 | 1,387 (7.4%) | 1.14 | 1.07 | 1.21 |
|  |  | Never | 4,007 | 564 (14.1%) | 1.50 | 1.37 | 1.64 |
|  | **No** | Daily | 15,284 | 1,458 (9.5%) | 1.17 | 1.10 | 1.25 |
|  |  | 2-4 times a week | 37,043 | 3,132 (8.5%) | 1.14 | 1.09 | 1.20 |
|  |  | Weekly | 49,346 | 3,578 (7.3%) | 1.12 | 1.07 | 1.18 |
|  |  | Monthly | 21,201 | 1,489 (7.0%) | 1.14 | 1.07 | 1.21 |
|  |  | Once every 3 months | 11,380 | 940 (8.3%) | 1.24 | 1.15 | 1.34 |
|  |  | Never | 3,547 | 491 (13.8%) | 1.49 | 1.36 | 1.64 |
|  |  |  |  |  |  |  |  |
| **CVD mortality** | **Yes** | Daily | 38,297 | 471 (1.2%) | 1 (ref) | - | - |
|  |  | 2-4 times a week | 104,838 | 1,077 (1.0%) | 0.96 | 0.86 | 1.07 |
|  |  | Weekly | 114,374 | 1,065 (0.9%) | 0.96 | 0.86 | 1.07 |
|  |  | Monthly | 40,183 | 385 (1.0%) | 1.01 | 0.88 | 1.15 |
|  |  | Once every 3 months | 18,646 | 243 (1.3%) | 1.18 | 1.01 | 1.38 |
|  |  | Never | 4,007 | 126 (3.1%) | 1.66 | 1.36 | 2.02 |
|  | **No** | Daily | 15,284 | 223 (1.5%) | 1.15 | 0.98 | 1.35 |
|  |  | 2-4 times a week | 37,043 | 447 (1.2%) | 1.05 | 0.92 | 1.20 |
|  |  | Weekly | 49,346 | 549 (1.1%) | 1.07 | 0.94 | 1.21 |
|  |  | Monthly | 21,201 | 242 (1.1%) | 1.10 | 0.94 | 1.29 |
|  |  | Once every 3 months | 11,380 | 175 (1.5%) | 1.30 | 1.09 | 1.55 |
|  |  | Never |  |  | 1.60 | 1.29 | 1.97 |

Models adjusted for sex, ethnicity, Townsend, month of assessment, smoking, alcohol, physical activity, BMI, long-term condition count, living alone, and functional isolation. HR, hazard ratio; lci, lower confidence interval; uci, upper confidence interval.

S7 Table

Interaction estimates for adverse health outcomes for binary exposures of frequency of friends and family visits (≥ monthly/< monthly) and weekly group activity (yes vs no).

| **All-cause mortality** | **Interaction** | **Estimate** |
| --- | --- | --- |
|  | Multiplicative scale | 0.94 [0.88, 1.01] |
|  | RERI | -0.05 [-0.14, 0.04] |
|  | AP | -0.04 [-0.11, 0.03] |
|  | SI | 0.86 [0.66, 1.13] |
|  | | |
| **CVD mortality** | Multiplicative scale | 0.96 [0.81, 1.14] |
|  | RERI | -0.02 [-0.24, 0.21] |
|  | AP | -0.01 [-0.19, 0.13 |
|  | SI | 0.96 [0.58, 1.60] |

Models adjusted for sex, ethnicity, Townsend, month of assessment, smoking, alcohol, physical activity, BMI, long-term condition count, living alone, and functional isolation. Estimates given as Hazard ratios [95% confidence intervals]). RERI, relative excess risk for interaction; AP, attributable portion, SI, synergy index. RERI or AP of zero means no interaction or perfect additivity. A RERI or AP of greater than zero means positive interaction or more than additivity. A RERI or AP of less than zero means negative interaction or less than additivity. An SI of greater than one means positive interaction or more than additivity. An SI of less than one means negative interaction or less than additivity.

S8 Table

Fully adjusted models of joint association between frequency of friends and family visits, living alone, and adverse health outcomes.

| **Outcome** | **Lives alone** | **Frequency of friends and family visits** | **N** | **Deaths (%)** | **HR** | **lci** | **uci** |
| --- | --- | --- | --- | --- | --- | --- | --- |
| **All-cause mortality** | **No** | Daily | 40,244 | 3,043 (7.6%) | 1 (ref) | - | - |
|  |  | 2-4 times a week | 113,223 | 7,726 (6.8%) | 0.98 | 0.94 | 1.02 |
|  |  | Weekly | 137,647 | 8,113 (5.9%) | 0.95 | 0.91 | 0.99 |
|  |  | Monthly | 52,781 | 3,137 (5.9%) | 1.00 | 0.95 | 1.05 |
|  |  | Once every 3 months | 24,725 | 1,662 (6.7%) | 1.07 | 1.01 | 1.14 |
|  |  | Never | 5,054 | 547 (10.8%) | 1.33 | 1.22 | 1.46 |
|  | **Yes** | Daily | 13,337 | 1,505 (11.3%) | 1.19 | 1.12 | 1.26 |
|  |  | 2-4 times a week | 28,658 | 2,765 (9.6%) | 1.16 | 1.10 | 1.22 |
|  |  | Weekly | 26,073 | 2,580 (9.9%) | 1.26 | 1.19 | 1.33 |
|  |  | Monthly | 8,603 | 884 (10.3%) | 1.30 | 1.20 | 1.40 |
|  |  | Once every 3 months | 5,301 | 665 (12.5%) | 1.43 | 1.31 | 1.56 |
|  |  | Never | 2,500 | 508 (20.3%) | 1.77 | 1.61 | 1.95 |
|  |  |  |  |  |  |  |  |
| **CVD mortality** | **No** | Daily | 40,244 | 441 (1.1%) | 1 (ref) | - | - |
|  |  | 2-4 times a week | 113,223 | 1,089 (1.0%) | 0.95 | 0.85 | 1.06 |
|  |  | Weekly | 137,647 | 1,165 (0.8%) | 0.91 | 0.81 | 1.01 |
|  |  | Monthly | 52,781 | 462 (0.9%) | 0.95 | 0.84 | 1.09 |
|  |  | Once every 3 months | 24,725 | 281 (1.1%) | 1.13 | 0.97 | 1.32 |
|  |  | Never | 5,054 | 109 (2.2%) | 1.49 | 1.21 | 1.84 |
|  | **Yes** | Daily | 13,337 | 253 (1.9%) | 1.39 | 1.19 | 1.63 |
|  |  | 2-4 times a week | 28,658 | 435 (1.5%) | 1.29 | 1.13 | 1.47 |
|  |  | Weekly | 26,073 | 449 (1.7%) | 1.45 | 1.27 | 1.66 |
|  |  | Monthly | 8,603 | 165 (1.9%) | 1.50 | 1.25 | 1.80 |
|  |  | Once every 3 months | 5,301 | 137 (2.6%) | 1.70 | 1.40 | 2.07 |
|  |  | Never | 2,500 | 126 (5.0%) | 2.23 | 1.82 | 2.73 |

Adjusted for sex, ethnicity, Townsend, month of assessment, smoking, alcohol, physical activity, BMI, long-term condition count, weekly group activity, and functional isolation. HR, hazard ratio; lci, lower confidence interval; uci, upper confidence interval.

S9 Table

| Interaction estimates for adverse health outcomes for binary exposures of frequency of friends and family visits (≥ monthly/< monthly) and living alone (yes/no). |
| --- |

|  | **Interaction** | **Estimate** |
| --- | --- | --- |
| All-cause mortality | Multiplicative scale | 1.11 [1.03, 1.20] |
|  | RERI | 0.20 [0.09, 0.31] |
|  | AP | 0.13 [0.06, 0.18] |
|  | SI | 1.15 [1.22, 1.87] |
|  | | |
| CVD mortality | Multiplicative scale | 1.07 [0.90, 1.27] |
|  | RERI | 0.27 [-0.01, 0.57] |
|  | AP | 0.13 [-0.01, 0.25] |
|  | SI | 1.35 [0.99, 1.86] |

Models adjusted for sex, ethnicity, Townsend, month of assessment, smoking, alcohol, physical activity, BMI, long-term condition count, weekly group activity, and functional isolation. Estimates given as Hazard ratios [95% confidence intervals]). RERI, relative excess risk for interaction; AP, attributable portion, SI, synergy index. RERI or AP of zero means no interaction or perfect additivity. A RERI or AP of greater than zero means positive interaction or more than additivity. A RERI or AP of less than zero means negative interaction or less than additivity. An SI of greater than one means positive interaction or more than additivity. An SI of less than one means negative interaction or less than additivity.

## S10 Table

Fully adjusted models of association between frequency of friends and family visits and adverse health outcomes stratified by living alone.

| Outcome | Lives alone | Frequency of friends and family visits | HR | lci | uci |
| --- | --- | --- | --- | --- | --- |
| All-cause mortality | No | Daily | 1 (ref) | - | - |
|  |  | 2-4 times a week | 0.98 | 0.94 | 1.02 |
|  |  | Weekly | 0.96 | 0.92 | 1.00 |
|  |  | Monthly | 1.01 | 0.96 | 1.07 |
|  |  | Once every 3 months | 1.09 | 1.02 | 1.16 |
|  |  | Never | 1.36 | 1.24 | 1.50 |
|  |  | | | | |
|  | Yes | Daily | 1 (ref) | - | - |
|  |  | 2-4 times a week | 0.97 | 0.91 | 1.03 |
|  |  | Weekly | 1.03 | 0.97 | 1.10 |
|  |  | Monthly | 1.05 | 0.97 | 1.15 |
|  |  | Once every 3 months | 1.15 | 1.05 | 1.26 |
|  |  | Never | 1.40 | 1.26 | 1.55 |
|  | | | | | |
| CVD mortality | No | Daily | 1 (ref) | - | - |
|  |  | 2-4 times a week | 0.96 | 0.85 | 1.07 |
|  |  | Weekly | 0.93 | 0.83 | 1.03 |
|  |  | Monthly | 0.98 | 0.86 | 1.12 |
|  |  | Once every 3 months | 1.16 | 1.00 | 1.35 |
|  |  | Never | 1.53 | 1.24 | 1.89 |
|  |  | | | | |
|  | Yes | Daily | 1 (ref) | - | - |
|  |  | 2-4 times a week | 0.91 | 0.78 | 1.07 |
|  |  | Weekly | 1.01 | 0.86 | 1.18 |
|  |  | Monthly | 1.03 | 0.85 | 1.26 |
|  |  | Once every 3 months | 1.16 | 0.94 | 1.43 |
|  |  | Never | 1.52 | 1.22 | 1.89 |

Models adjusted for sex, ethnicity, Townsend, month of assessment, smoking, alcohol, physical activity, BMI, long-term condition count, weekly group activity, and functional isolation. HR, hazard ratio; lci, lower confidence interval; uci, upper confidence interval.

## S11 Table

Fully adjusted models of joint association between weekly group activity, living alone, and adverse health outcomes.

| Outcome | Lives alone | Weekly group activity | N | Deaths (%) | HR | lci | uci |
| --- | --- | --- | --- | --- | --- | --- | --- |
| All-cause mortality | No | Yes | 260,452 | 16,238 (6.2) | 1 (ref) |  |  |
|  |  | No | 113,222 | 7,990 (7.1) | 1.11 | 1.08 | 1.14 |
|  | Yes | Yes | 59,893 | 5,809 (9.7) | 1.23 | 1.19 | 1.26 |
|  |  | No | 24,579 | 3,098 (12.6) | 1.46 | 1.40 | 1.52 |
|  |  |  |  |  |  |  |  |
| CVD mortality | No | Yes | 260,452 | 2,358 (0.9) | 1 (ref) |  |  |
|  |  | No | 113,222 | 1,189 (1.1) | 1.08 | 1.01 | 1.16 |
|  | Yes | Yes | 59,893 | 1,009 (1.7) | 1.45 | 1.34 | 1.57 |
|  |  | No | 24,579 | 556 (2.3) | 1.66 | 1.50 | 1.83 |

Models adjusted for sex, ethnicity, Townsend, month of assessment, smoking, alcohol, physical activity, BMI, long-term condition count, frequency of friends and family visits, and functional isolation. HR, hazard ratio; lci, lower confidence interval; uci, upper confidence interval.

## S12 Table

Interaction estimates for adverse health outcomes for binary exposures of weekly group activity (yes/no) and living alone (yes/no).

|  | **Interaction** | **Estimate** |
| --- | --- | --- |
| All-cause mortality | Multiplicative scale | 1.07 [1.02, 1.13] |
|  | RERI | 0.12 [0.06, 0.19] |
|  | AP | 0.08 [0.04, 0.13] |
|  | SI | 1.37 [1.15, 1.63] |
|  | | |
| CVD mortality | Multiplicative scale | 1.05 [0.93, 1.19] |
|  | RERI | 0.12 [-0.06, 0.3] |
|  | AP | 0.07 [-0.04, 0.17] |
|  | SI | 1.23 [0.90, 1.67] |

Models adjusted for sex, ethnicity, Townsend, month of assessment, smoking, alcohol, physical activity, BMI, long-term condition count, frequency of friends and family visits, and functional isolation. Estimates given as Hazard ratios [95% confidence intervals]). RERI, relative excess risk for interaction; AP, attributable portion, SI, synergy index. RERI or AP of zero means no interaction or perfect additivity. A RERI or AP of greater than zero means positive interaction or more than additivity. A RERI or AP of less than zero means negative interaction or less than additivity. An SI of greater than one means positive interaction or more than additivity. An SI of less than one means negative interaction or less than additivity.

## S13 Table

Fully adjusted models of association between weekly group activity and adverse health outcomes stratified by living alone.

| Outcome | Living alone | Weekly group activity | N | deaths (%) | HR | lci | uci |
| --- | --- | --- | --- | --- | --- | --- | --- |
| All-cause mortality | No | Yes | 260,452 | 16,238 (6.2) | 1 (ref) |  |  |
|  |  | No | 113,222 | 7,990 (7.1) | 1.11 | 1.08 | 1.14 |
|  |  |  |  |  |  |  |  |
|  | Yes | Yes | 59,893 | 5,809 (9.7) | 1 (ref) |  |  |
|  |  | No | 24,579 | 3,098 (12.6) | 1.19 | 1.14 | 1.25 |
|  |  |  |  |  |  |  |  |
| CVD mortality | No | Yes | 260,452 | 2,358 (0.9) | 1 (ref) |  |  |
|  |  | No | 113,222 | 1,189 (1.1) | 1.08 | 1.00 | 1.16 |
|  |  |  |  |  |  |  |  |
|  | Yes | Yes | 59,893 | 1,009 (1.7) | 1 (ref) |  |  |
|  |  | No | 24,579 | 556 (2.3) | 1.16 | 1.04 | 1.29 |

Models adjusted for sex, ethnicity, Townsend, month of assessment, smoking, alcohol, physical activity, BMI, long-term condition count, frequency of friends and family visits, and functional isolation. HR, hazard ratio; lci, lower confidence interval; uci, upper confidence interval.

## S14 Table

Fully adjusted models of joint associations between frequency of ability to confide in someone close, often feeling lonely, and structural isolation (defined as <monthly friends and family visits or not engaging in weekly group activity or living alone), and adverse health outcomes. Models adjusted for sex, ethnicity, Townsend, month of assessment, smoking, alcohol, physical activity, BMI, long-term condition count. HR, hazard ratio; LCI, lower confidence interval; UCI, upper confidence interval.

### **All-cause mortality**

| Weekly group activity, living alone, and structural isolation group | Frequency of ability to confide | N | deaths (%) | HR | lci | uci |
| --- | --- | --- | --- | --- | --- | --- |
| - not often lonely - no structural isolation | Daily | 142,049 | 8,693 (6.1%) | 1.00 |  |  |
|  | 2-4 times a week | 19,876 | 1,035 (5.2%) | 0.98 | 0.92 | 1.05 |
|  | Weekly | 20,142 | 1,162 (5.8%) | 0.99 | 0.94 | 1.06 |
|  | Monthly | 9,460 | 579 (6.1%) | 1.04 | 0.96 | 1.13 |
|  | Once every 3 months | 10,725 | 667 (6.2%) | 0.98 | 0.90 | 1.06 |
|  | Never | 22,971 | 1,869 (8.1%) | 1.07 | 1.02 | 1.12 |
| - not often lonely - structural isolation | Daily | 79,906 | 6,022 (7.5%) | 1.18 | 1.14 | 1.22 |
|  | 2-4 times a week | 14,912 | 1,062 (7.1%) | 1.19 | 1.12 | 1.27 |
|  | Weekly | 16,620 | 1,330 (8.0%) | 1.23 | 1.16 | 1.30 |
|  | Monthly | 7,549 | 580 (7.7%) | 1.14 | 1.05 | 1.24 |
|  | Once every 3 months | 8,073 | 701 (8.7%) | 1.24 | 1.15 | 1.34 |
|  | Never | 21,948 | 2,482 (11.3%) | 1.38 | 1.32 | 1.44 |
| - often lonely - no structural isolation | Daily | 12,002 | 752 (6.3%) | 1.15 | 1.06 | 1.24 |
|  | 2-4 times a week | 3,927 | 228 (5.8%) | 1.23 | 1.07 | 1.40 |
|  | Weekly | 5,331 | 315 (5.9%) | 1.14 | 1.02 | 1.28 |
|  | Monthly | 2,966 | 170 (5.7%) | 1.13 | 0.97 | 1.31 |
|  | Once every 3 months | 2,810 | 156 (5.6%) | 1.05 | 0.89 | 1.23 |
|  | Never | 8,193 | 612 (7.5%) | 1.16 | 1.07 | 1.26 |
| - often lonely - structural isolation | Daily | 12,894 | 1,121 (8.7%) | 1.27 | 1.19 | 1.35 |
|  | 2-4 times a week | 5,552 | 462 (8.3%) | 1.25 | 1.14 | 1.38 |
|  | Weekly | 8,227 | 749 (9.1%) | 1.30 | 1.21 | 1.40 |
|  | Monthly | 4,428 | 437 (9.9%) | 1.40 | 1.27 | 1.54 |
|  | Once every 3 months | 4,059 | 369 (9.1%) | 1.29 | 1.17 | 1.44 |
|  | Never | 13,526 | 1,582 (11.7%) | 1.41 | 1.34 | 1.49 |

### **CVD mortality**

| Weekly group activity, living alone, and functional isolation group | Frequency of ability to confide | N | deaths (%) | HR | lci | uci |
| --- | --- | --- | --- | --- | --- | --- |
| - not often lonely - no structural isolation | Daily | 142,049 | 1,248 (0.9%) | 1.00 |  |  |
|  | 2-4 times a week | 19,876 | 128 (0.6%) | 0.92 | 0.76 | 1.10 |
|  | Weekly | 20,142 | 151 (0.7%) | 0.97 | 0.82 | 1.15 |
|  | Monthly | 9,460 | 81 (0.9%) | 1.09 | 0.87 | 1.37 |
|  | Once every 3 months | 10,725 | 99 (0.9%) | 1.03 | 0.84 | 1.27 |
|  | Never | 22,971 | 311 (1.4%) | 1.15 | 1.01 | 1.30 |
| - not often lonely - structural isolation | Daily | 79,906 | 887 (1.1%) | 1.20 | 1.10 | 1.31 |
|  | 2-4 times a week | 14,912 | 156 (1.0%) | 1.33 | 1.12 | 1.57 |
|  | Weekly | 16,620 | 194 (1.2%) | 1.34 | 1.15 | 1.56 |
|  | Monthly | 7,549 | 84 (1.1%) | 1.17 | 0.94 | 1.47 |
|  | Once every 3 months | 8,073 | 112 (1.4%) | 1.40 | 1.16 | 1.70 |
|  | Never | 21,948 | 481 (2.2%) | 1.67 | 1.50 | 1.86 |
| - often lonely - no structural isolation | Daily | 12,002 | 117 (1.0%) | 1.23 | 1.02 | 1.49 |
|  | 2-4 times a week | 3,927 | 22 (0.6%) | 0.87 | 0.57 | 1.33 |
|  | Weekly | 5,331 | 41 (0.8%) | 1.14 | 0.83 | 1.55 |
|  | Monthly | 2,966 | 23 (0.8%) | 1.15 | 0.76 | 1.73 |
|  | Once every 3 months | 2,810 | 31 (1.1%) | 1.53 | 1.07 | 2.19 |
|  | Never | 8,193 | 106 (1.3%) | 1.31 | 1.07 | 1.60 |
| - often lonely - structural isolation | Daily | 12,894 | 173 (1.3%) | 1.34 | 1.14 | 1.58 |
|  | 2-4 times a week | 5,552 | 74 (1.3%) | 1.46 | 1.15 | 1.85 |
|  | Weekly | 8,227 | 118 (1.4%) | 1.43 | 1.19 | 1.74 |
|  | Monthly | 4,428 | 84 (1.9%) | 1.91 | 1.53 | 2.38 |
|  | Once every 3 months | 4,059 | 58 (1.4%) | 1.39 | 1.07 | 1.82 |
|  | Never | 13,526 | 333 (2.5%) | 1.78 | 1.57 | 2.01 |

### S2 Figure


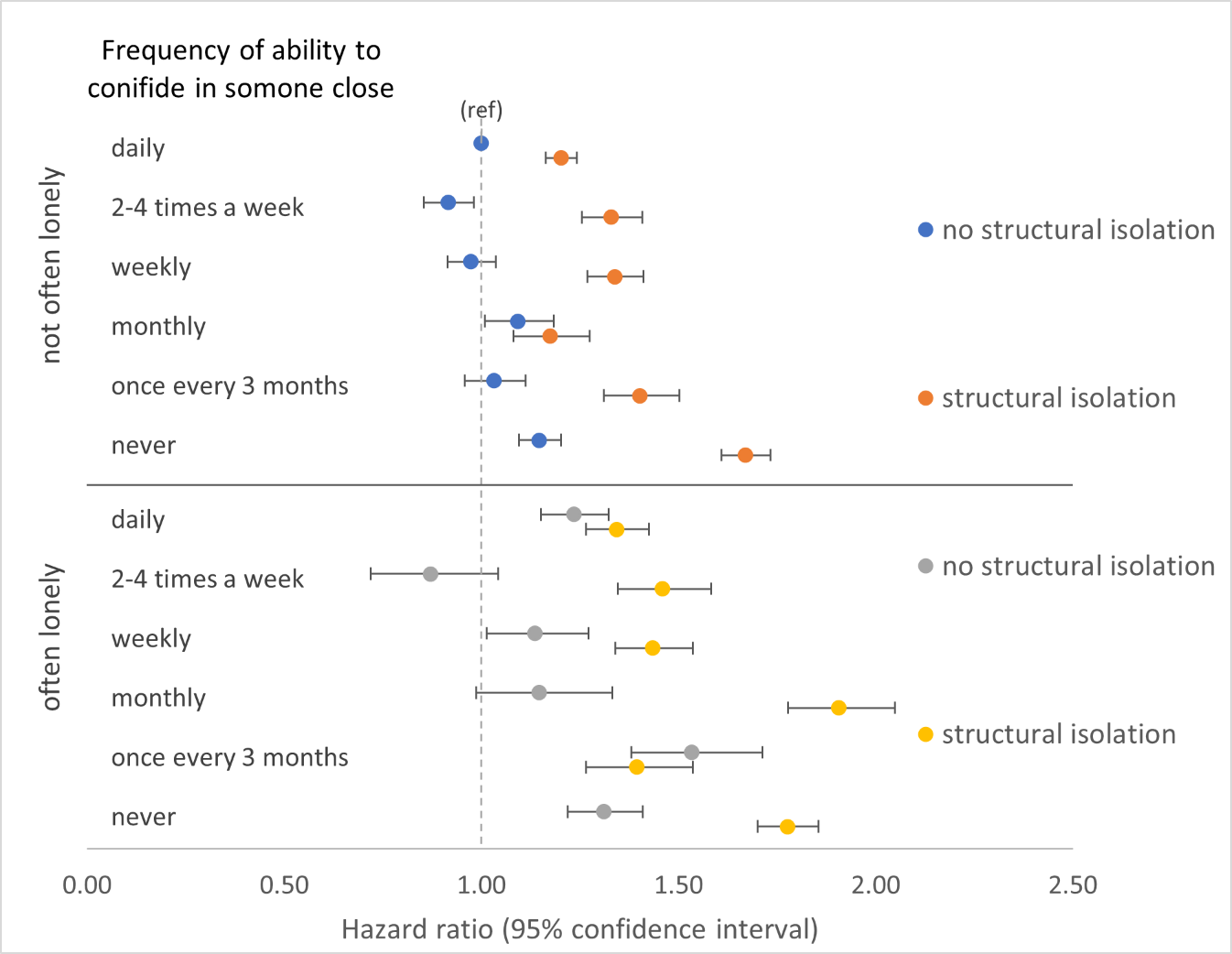


Figure S2 Models of joint association between frequency of ability to confide in someone close, often feeling lonely, structural isolation, and CVD mortality.

## S15 Table

Fully adjusted models of joint associations between frequency of friends and family visits, weekly group activity, living alone, functional isolation (defined as either never able to confide in someone close or often feeling lonely), and adverse health outcomes. Models adjusted for sex, ethnicity, Townsend, month of assessment, smoking, alcohol, physical activity, BMI, long-term condition count. HR, hazard ratio; LCI, lower confidence interval; UCI, upper confidence interval.

### **All-cause mortality**

| Weekly group activity, living alone, and functional isolation category | Frequency of friends and family visits | N | deaths (%) | HR | lci | uci |
| --- | --- | --- | --- | --- | --- | --- |
| - weekly group activity - not living alone - no functional isolation | Daily | 23,052 | 1,626 (7.1%) | 1 (ref) | - | - |
|  | 2-4 times a week | 67,443 | 4,280 (6.3%) | 0.98 | 0.93 | 1.04 |
|  | Weekly | 74,356 | 4,018 (5.4%) | 0.95 | 0.90 | 1.01 |
|  | Monthly | 25,741 | 1,404 (5.5%) | 1.01 | 0.94 | 1.09 |
|  | Once every 3 months | 10,386 | 660 (6.4%) | 1.11 | 1.01 | 1.22 |
|  | Never | 1,274 | 148 (11.6%) | 1.57 | 1.32 | 1.85 |
| - weekly group activity - living alone - no functional isolation | Daily | 6,167 | 584 (9.5%) | 1.14 | 1.04 | 1.25 |
|  | 2-4 times a week | 12,963 | 1,048 (8.1%) | 1.16 | 1.07 | 1.25 |
|  | Weekly | 9,728 | 791 (8.1%) | 1.25 | 1.15 | 1.36 |
|  | Monthly | 2,790 | 219 (7.8%) | 1.24 | 1.08 | 1.43 |
|  | Once every 3 months | 1,362 | 141 (10.4%) | 1.53 | 1.28 | 1.81 |
|  | Never | 279 | 54 (19.4%) | 2.21 | 1.68 | 2.90 |
| - weekly group activity - not living alone - functional isolation | Daily | 5,492 | 459 (8.4%) | 1.14 | 1.03 | 1.26 |
|  | 2-4 times a week | 15,932 | 1,165 (7.3%) | 1.05 | 0.97 | 1.13 |
|  | Weekly | 21,779 | 1,414 (6.5%) | 1.07 | 0.99 | 1.14 |
|  | Monthly | 8,775 | 586 (6.7%) | 1.17 | 1.06 | 1.28 |
|  | Once every 3 months | 4,863 | 343 (7.1%) | 1.21 | 1.08 | 1.36 |
|  | Never | 1,359 | 135 (9.9%) | 1.43 | 1.20 | 1.70 |
| - weekly group activity - living alone - functional isolation | Daily | 3,586 | 421 (11.7%) | 1.26 | 1.13 | 1.41 |
|  | 2-4 times a week | 8,500 | 866 (10.2%) | 1.23 | 1.13 | 1.33 |
|  | Weekly | 8,511 | 892 (10.5%) | 1.37 | 1.26 | 1.49 |
|  | Monthly | 2,877 | 323 (11.2%) | 1.46 | 1.30 | 1.65 |
|  | Once every 3 months | 2,035 | 243 (11.9%) | 1.45 | 1.27 | 1.66 |
|  | Never | 1,095 | 227 (20.7%) | 1.98 | 1.72 | 2.27 |
| - no weekly group activity - not living alone - no functional isolation | Daily | 8,718 | 699 (8.0%) | 1.14 | 1.04 | 1.24 |
|  | 2-4 times a week | 22,718 | 1,695 (7.5%) | 1.14 | 1.07 | 1.22 |
|  | Weekly | 30,465 | 1,853 (6.1%) | 1.07 | 1.00 | 1.14 |
|  | Monthly | 12,859 | 779 (6.1%) | 1.11 | 1.02 | 1.21 |
|  | Once every 3 months | 6,008 | 395 (6.6%) | 1.16 | 1.04 | 1.29 |
|  | Never | 1,021 | 118 (11.6%) | 1.55 | 1.29 | 1.87 |
| - no weekly group activity - living alone - no functional isolation | Daily | 1,977 | 249 (12.6%) | 1.49 | 1.31 | 1.71 |
|  | 2-4 times a week | 3,943 | 406 (10.3%) | 1.40 | 1.25 | 1.56 |
|  | Weekly | 3,892 | 398 (10.2%) | 1.50 | 1.34 | 1.67 |
|  | Monthly | 1,292 | 138 (10.7%) | 1.61 | 1.35 | 1.91 |
|  | Once every 3 months | 708 | 95 (13.4%) | 1.73 | 1.41 | 2.13 |
|  | Never | 170 | 33 (19.4%) | 2.34 | 1.65 | 3.30 |
| - no weekly group activity - not living alone - functional isolation | Daily | 2,982 | 259 (8.7%) | 1.22 | 1.07 | 1.40 |
|  | 2-4 times a week | 7,130 | 586 (8.2%) | 1.17 | 1.06 | 1.28 |
|  | Weekly | 11,047 | 828 (7.5%) | 1.20 | 1.10 | 1.31 |
|  | Monthly | 5,406 | 368 (6.8%) | 1.16 | 1.03 | 1.29 |
|  | Once every 3 months | 3,468 | 264 (7.6%) | 1.27 | 1.11 | 1.45 |
|  | Never | 1,400 | 146 (10.4%) | 1.44 | 1.21 | 1.71 |
| - no weekly group activity - living alone - functional isolation | Daily | 1,607 | 251 (15.6%) | 1.58 | 1.38 | 1.80 |
|  | 2-4 times a week | 3,252 | 445 (13.7%) | 1.49 | 1.34 | 1.65 |
|  | Weekly | 3,942 | 499 (12.7%) | 1.55 | 1.40 | 1.71 |
|  | Monthly | 1,644 | 204 (12.4%) | 1.55 | 1.34 | 1.79 |
|  | Once every 3 months | 1,196 | 186 (15.6%) | 1.82 | 1.56 | 2.12 |
|  | Never | 956 | 194 (20.3%) | 1.99 | 1.71 | 2.31 |

### **CVD mortality**

| Weekly group activity, living alone, and functional isolation category | Frequency of friends and family visits | N | deaths (%) | HR | LCI | UCI |
| --- | --- | --- | --- | --- | --- | --- |
| - weekly group activity - not living alone - no functional isolation | Daily | 23,052 | 226 (1.0%) | 1.00 |  |  |
|  | 2-4 times a week | 67,443 | 596 (0.9%) | 0.98 | 0.84 | 1.14 |
|  | Weekly | 74,356 | 565 (0.8%) | 0.93 | 0.80 | 1.09 |
|  | Monthly | 25,741 | 190 (0.7%) | 0.93 | 0.77 | 1.13 |
|  | Once every 3 months | 10,386 | 103 (1.0%) | 1.14 | 0.90 | 1.44 |
|  | Never | 1,274 | 27 (2.1%) | 1.70 | 1.14 | 2.54 |
| - weekly group activity - lives alone - no functional isolation | Daily | 6,167 | 95 (1.5%) | 1.43 | 1.13 | 1.82 |
|  | 2-4 times a week | 12,963 | 144 (1.1%) | 1.25 | 1.01 | 1.54 |
|  | Weekly | 9,728 | 119 (1.2%) | 1.39 | 1.11 | 1.74 |
|  | Monthly | 2,790 | 37 (1.3%) | 1.48 | 1.04 | 2.09 |
|  | Once every 3 months | 1,362 | 24 (1.8%) | 1.74 | 1.14 | 2.65 |
|  | Never | 279 | 11 (3.9%) | 2.72 | 1.48 | 4.99 |
| - weekly group activity - not living alone - functional isolation | Daily | 5,492 | 74 (1.3%) | 1.22 | 0.94 | 1.58 |
|  | 2-4 times a week | 15,932 | 191 (1.2%) | 1.17 | 0.97 | 1.42 |
|  | Weekly | 21,779 | 208 (1.0%) | 1.05 | 0.87 | 1.27 |
|  | Monthly | 8,775 | 89 (1.0%) | 1.18 | 0.92 | 1.50 |
|  | Once every 3 months | 4,863 | 61 (1.3%) | 1.40 | 1.05 | 1.86 |
|  | Never | 1,359 | 28 (2.1%) | 1.76 | 1.19 | 2.60 |
| - weekly group activity - living alone - functional isolation | Daily | 3,586 | 76 (2.1%) | 1.56 | 1.20 | 2.02 |
|  | 2-4 times a week | 8,500 | 146 (1.7%) | 1.45 | 1.18 | 1.79 |
|  | Weekly | 8,511 | 173 (2.0%) | 1.79 | 1.46 | 2.18 |
|  | Monthly | 2,877 | 69 (2.4%) | 1.98 | 1.51 | 2.59 |
|  | Once every 3 months | 2,035 | 55 (2.7%) | 1.98 | 1.47 | 2.66 |
|  | Never | 1,095 | 60 (5.5%) | 2.80 | 2.10 | 3.74 |
| - no weekly group activity - not living alone - no functional isolation | Daily | 8,718 | 96 (1.1%) | 1.11 | 0.87 | 1.40 |
|  | 2-4 times a week | 22,718 | 228 (1.0%) | 1.07 | 0.89 | 1.29 |
|  | Weekly | 30,465 | 263 (0.9%) | 1.02 | 0.85 | 1.22 |
|  | Monthly | 12,859 | 113 (0.9%) | 1.04 | 0.83 | 1.31 |
|  | Once every 3 months | 6,008 | 71 (1.2%) | 1.32 | 1.01 | 1.73 |
|  | Never | 1,021 | 30 (2.9%) | 2.26 | 1.54 | 3.31 |
| - no weekly group activity - living alone - no functional isolation | Daily | 1,977 | 37 (1.9%) | 1.72 | 1.22 | 2.44 |
|  | 2-4 times a week | 3,943 | 64 (1.6%) | 1.69 | 1.28 | 2.23 |
|  | Weekly | 3,892 | 61 (1.6%) | 1.62 | 1.22 | 2.15 |
|  | Monthly | 1,292 | 17 (1.3%) | 1.34 | 0.81 | 2.19 |
|  | Once every 3 months | 708 | 18 (2.5%) | 2.04 | 1.26 | 3.30 |
|  | Never | 170 | 5 (2.9%) | 2.12 | 0.87 | 5.14 |
| - no weekly group activity - not living alone - functional isolation | Daily | 2,982 | 45 (1.5%) | 1.45 | 1.05 | 2.00 |
|  | 2-4 times a week | 7,130 | 74 (1.0%) | 1.00 | 0.77 | 1.30 |
|  | Weekly | 11,047 | 129 (1.2%) | 1.21 | 0.97 | 1.50 |
|  | Monthly | 5,406 | 70 (1.3%) | 1.40 | 1.07 | 1.83 |
|  | Once every 3 months | 3,468 | 46 (1.3%) | 1.37 | 0.99 | 1.88 |
|  | Never | 1,400 | 24 (1.7%) | 1.36 | 0.89 | 2.08 |
| - no weekly group activity - living alone - functional isolation | Daily | 1,607 | 45 (2.8%) | 1.83 | 1.32 | 2.52 |
|  | 2-4 times a week | 3,252 | 81 (2.5%) | 1.84 | 1.42 | 2.37 |
|  | Weekly | 3,942 | 96 (2.4%) | 1.92 | 1.51 | 2.45 |
|  | Monthly | 1,644 | 42 (2.6%) | 1.92 | 1.38 | 2.68 |
|  | Once every 3 months | 1,196 | 40 (3.3%) | 2.23 | 1.59 | 3.12 |
|  | Never | 956 | 50 (5.2%) | 2.67 | 1.96 | 3.65 |

### S3 Figure


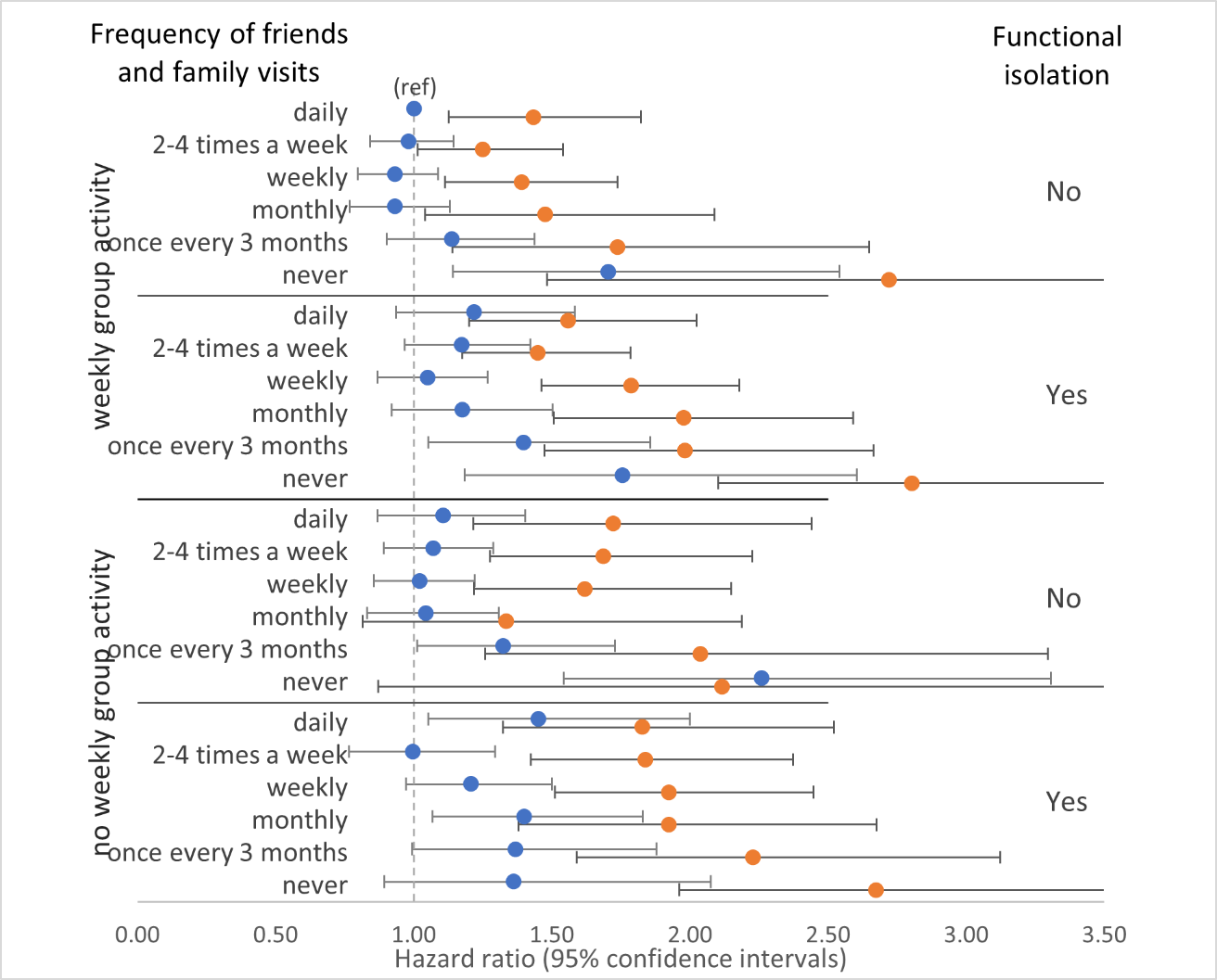


Figure S3 Models of joint association between frequency of friends and family visits, weekly group activity, living alone, functional isolation, and CVD mortality.

S16 Table

Fully adjusted models of joint association between functional and structural isolation and adverse health outcomes.

| Outcome | Functional isolation | Structural isolation | N | Deaths (%) | HR | lci | uci |
| --- | --- | --- | --- | --- | --- | --- | --- |
| All-cause mortality | No | No | 190,592 | 11,328 (5.9%) | 1 (ref) | - | - |
|  | Yes | No | 51,978 | 3,624 (7.0%) | 1.11 | 1.06 | 1.15 |
|  | No | Yes | 138,720 | 10,503 (7.6%) | 1.21 | 1.17 | 1.24 |
|  | Yes | Yes | 76,856 | 7,680 (10.0%) | 1.36 | 1.32 | 1.40 |
|  |  |  |  |  |  |  |  |
| CVD mortality | No | No | 190,592 | 1,577 (0.8%) | 1 (ref) | - | - |
|  | Yes | No | 51,978 | 562 (1.1%) | 1.17 | 1.06 | 1.29 |
|  | No | Yes | 138,720 | 1,563 (1.1%) | 1.27 | 1.18 | 1.36 |
|  | Yes | Yes | 76,856 | 1,410 (1.8%) | 1.63 | 1.51 | 1.76 |

S17 Table

Interaction estimates for adverse health outcomes for binary exposures of functional and structural isolation.

|  | **Interaction** | **Estimate** |
| --- | --- | --- |
| All-cause mortality | Multiplicative scale | 1.02 [0.97, 1.07] |
|  | RERI | 0.05 [-0.01, 0.1] |
|  | AP | 0.03 [-0.01, 0.07] |
|  | SI | 1.15 [0.97, 1.37] |
|  | | |
| CVD mortality | Multiplicative scale | 1.10 [0.97, 1.24] |
|  | RERI | 0.19 [0.04, 0.34] |
|  | AP | 0.12 [0.02, 0.2] |
|  | SI | 1.43 [1.03, 1.99] |

Models adjusted for sex, ethnicity, Townsend, month of assessment, smoking, alcohol, physical activity, BMI, long-term condition count. Estimates given as Hazard ratios [95% confidence intervals]). RERI, relative excess risk for interaction; AP, attributable portion, SI, synergy index. RERI or AP of zero means no interaction or perfect additivity. A RERI or AP of greater than zero means positive interaction or more than additivity. A RERI or AP of less than zero means negative interaction or less than additivity. An SI of greater than one means positive interaction or more than additivity. An SI of less than one means negative interaction or less than additivity.
