# Supplementary material for: Social connection and mortality in UK Biobank: a prospective cohort analysis
Source: BMC Med. 2023 Nov 10;21:384. doi: 10.1186/s12916-023-03055-7 (PMC10637015; doi:10.1186/s12916-023-03055-7)
Supplement: Supplementary file 2 — Additional file 2: Tables S18-S35 show results from sensitivity analyses where those with self-reported prior CVD or cancer or who died with 2 years of recruitment were excluded. Table S18. Associations between frequency of ability to confide in someone close and adverse health outcomes. Table S19. Associations between often feeling lonely and adverse health outcomes. Table S20. Combined associations between frequency of ability to confide in someone close, often feeling lonely and adverse health outcomes. Table S21. Interaction estimates for adverse health outcomes for binary exposures of never able to confide in someone close and often feeling lonely. Table S22. Associations between functional isolation and all-cause and CVD mortality. Table S23. Associations between structural component measures and all-cause and CVD mortality. Table S24. Combined associations between frequency of friends and family visits, engaging in weekly group activity, and adverse health outcomes. Table S25. Interaction estimates for adverse health outcomes for binary exposures of frequency of friends and family visits and weekly group activity. Table S26. Combined associations between frequency of friends and family visits, living alone, and adverse health outcomes. Table S27. Interaction estimates for adverse health outcomes for binary exposures of friends and family visits less than monthly and living alone. Table S28. Associations between frequency of friends and family visits and adverse health outcomes stratified by living alone. Table S29. Combined associations between weekly group activity, living alone, and adverse health outcomes. Table S30. Interaction estimates for adverse health outcomes for binary exposures of weekly group activity and living alone. Table S31. Associations between weekly group activity and adverse health outcomes stratified by living alone. Table S32. Combined associations between frequency of ability to confide in someone close, often feeling lonely, and stru [file 12916_2023_3055_MOESM2_ESM.docx]

# Additional File 2

## Sensitivity analyses

Similar results were seen across all sensitivity analyses where we excluded those with prior CVD or cancer or who died within 2 years of recruitment (Tables S18-S35). For example, the associations between functional isolation and all-cause and CVD mortality were 1.12 (1.07-1.16) and 1.18 (1.05-1.32), respectively (Table S22). And compared to those with daily friends and family visits, those who never had friends or family visits hazards for all-cause and CVD mortality were 1.43 (1.27-1.61) and 1.58 (1.17-2.12), respectively (Table S23). A significant interaction on a multiplicative scale between frequency of friends and family visits and living alone for all-cause mortality was observed once again (Table S27) with markedly stronger associations with each of the adverse health outcomes at every level of friend and family visit frequency in those who lived alone (Table S26). For example, all-cause mortality HRs for those who reported never having friends and family visits were 1.20 (1.02-1.42) in those not living alone and 2.09 (1.77-2.47) in those living alone. As per main analyses, results were suggestive of an additive interaction between frequency of friends and family visits and living alone for CVD-mortality, but the estimates had wide confidence intervals (Tables S26 & S27). Also consistent with the main results were the results from sensitivity analyses examining the interaction between weekly group activity and living alone (a multiplicative interaction for all-cause mortality and strongly suggestive of an additive interaction for CVD-mortality) interaction. Results were also consistent with an additive interaction between overall functional and structural isolation (Tables S28 and S29).

S18 Table

Fully adjusted models of association between frequency of ability to confide in someone close and adverse health outcomes. Excluding those with self-reported prior CVD or cancer or who died with 2 years of recruitment. Participants included in analysis n = 292,516. After median follow up of 12.6 years (IQR 11.9-13.3) there were 12,574 (4.3%) deaths including 1,462 (0.5%) CVD deaths).

| Outcome | Frequency of ability to confide in someone close | N | Deaths (%) | HR | lci | uci |  |
| --- | --- | --- | --- | --- | --- | --- | --- |
| All-cause mortality | Daily | 159,873 | 6,326 (4.0%) | 1 (ref) |  |  |  |
|  | 2-4 times a week | 29,207 | 1,099 (3.8%) | 1.00 | 0.94 | 1.07 |  |
|  | Weekly | 32,436 | 1,428 (4.4%) | 1.05 | 0.99 | 1.12 |  |
|  | Monthly | 15,586 | 686 (4.4%) | 1.05 | 0.97 | 1.13 |  |
|  | Once every 3 months | 16,036 | 706 (4.4%) | 0.99 | 0.91 | 1.07 |  |
|  | Never | 39,378 | 2,329 (5.9%) | 1.11 | 1.06 | 1.17 |  |
|  | | | | | | | |
| CVD mortality | Daily | 159,873 | 727 (0.5%) | 1 (ref) |  |  |  |
|  | 2-4 times a week | 29,207 | 112 (0.4%) | 0.91 | 0.74 | 1.12 |  |
|  | Weekly | 32,436 | 159 (0.5%) | 1.02 | 0.85 | 1.22 |  |
|  | Monthly | 15,586 | 70 (0.4%) | 0.92 | 0.71 | 1.17 |  |
|  | Once every 3 months | 16,036 | 70 (0.4%) | 0.81 | 0.63 | 1.04 |  |
|  | Never | 39,378 | 324 (0.8%) | 1.13 | 0.98 | 1.30 |  |

Model adjusted for sex, ethnicity, Townsend, month of assessment, smoking, alcohol, physical activity, BMI, long-term condition count, frequency of friend and family visits, weekly group activity, living alone, and often feeling lonely. HR, hazard ratio; lci, lower confidence interval; uci, upper confidence interval.

S19 Table

Fully adjusted models of association between often feeling lonely and adverse health outcomes. Those with self-reported prior CVD or cancer or who died with 2 years of recruitment excluded.

| Outcome | Often feels lonely | N | Deaths (%) | HR | lci | uci |
| --- | --- | --- | --- | --- | --- | --- |
| All-cause mortality | No | 241,292 | 10,187 (4.2%) | 1 (ref) |  |  |
|  | Yes | 51,224 | 2,387 (4.7%) | 1.08 | 1.03 | 1.13 |
| CVD mortality | No | 241,292 | 1,157 (0.5%) | 1 (ref) |  |  |
|  | Yes | 51,224 | 305 (0.6%) | 1.17 | 1.02 | 1.35 |

Models adjusted for sex, ethnicity, Townsend, month of assessment, smoking, alcohol, physical activity, BMI, long-term condition count, frequency of friend and family visits, weekly group activity, living alone, and frequency of ability to confide. HR, hazard ratio; lci, lower confidence interval; uci, upper confidence interval.

S20 Table

Fully adjusted models of combined associations between frequency of ability to confide in someone close, often feeling lonely and adverse health outcomes. Those with self-reported prior CVD or cancer or who died with 2 years of recruitment excluded.

| Outcome | Often feels lonely | Frequency of ability to confide in someone close | HR | lci | uci |
| --- | --- | --- | --- | --- | --- |
| All-cause mortality | No | Daily | 1 (ref) | - | - |
|  |  | 2-4 times a week | 0.99 | 0.92 | 1.06 |
|  |  | Weekly | 1.04 | 0.98 | 1.12 |
|  |  | Monthly | 1.08 | 0.98 | 1.18 |
|  |  | Once every 3 months | 0.98 | 0.90 | 1.08 |
|  |  | Never | 1.13 | 1.07 | 1.19 |
|  | Yes | Daily | 1.09 | 1.01 | 1.19 |
|  |  | 2-4 times a week | 1.14 | 1.00 | 1.29 |
|  |  | Weekly | 1.16 | 1.05 | 1.29 |
|  |  | Monthly | 1.05 | 0.91 | 1.22 |
|  |  | Once every 3 months | 1.09 | 0.94 | 1.26 |
|  |  | Never | 1.16 | 1.07 | 1.26 |
|  |  |  |  |  |  |
| CVD mortality | No | Daily | 1 (ref) | - | - |
|  |  | 2-4 times a week | 0.88 | 0.70 | 1.11 |
|  |  | Weekly | 1.03 | 0.84 | 1.26 |
|  |  | Monthly | 0.93 | 0.69 | 1.24 |
|  |  | Once every 3 months | 0.81 | 0.60 | 1.08 |
|  |  | Never | 1.11 | 0.94 | 1.30 |
|  | Yes | Daily | 1.12 | 0.88 | 1.43 |
|  |  | 2-4 times a week | 1.16 | 0.79 | 1.69 |
|  |  | Weekly | 1.16 | 0.85 | 1.57 |
|  |  | Monthly | 1.03 | 0.67 | 1.59 |
|  |  | Once every 3 months | 0.95 | 0.60 | 1.49 |
|  |  | Never | 1.35 | 1.09 | 1.67 |

Models adjusted for sex, ethnicity, Townsend, month of assessment, smoking, alcohol, physical activity, BMI, long-term condition count, frequency of friend and family visits, weekly group activity, living alone. HR, hazard ratio; lci, lower confidence interval; uci, upper confidence interval.

S21 Table

Interaction estimates for adverse health outcomes for binary exposures of never able to confide in someone close and often feeling lonely. Those with self-reported prior CVD or cancer or who died with 2 years of recruitment excluded.

|  | **Interaction** | **Estimate** |
| --- | --- | --- |
| All-cause mortality | Multiplicative scale | 0.95 [0.86, 1.05] |
|  | RERI | -0.05 [-0.17, 0.07] |
|  | AP | -0.04 [-0.15, 0.05] |
|  | SI | 0.80 [0.47, 1.38] |
|  | | |
| CVD mortality | Multiplicative scale | 1.11 [0.84, 1.47] |
|  | RERI | 0.17 [-0.18, 0.55] |
|  | AP | 0.12 [-0.16, 0.30] |
|  | SI | 1.55 [0.60, 1.05] |

Models adjusted for sex, ethnicity, Townsend, month of assessment, smoking, alcohol, physical activity, BMI, long-term condition count, frequency of friend and family visits, weekly group activity, living alone. Estimates given as Hazard ratios [95% confidence intervals]). RERI, relative excess risk for interaction; AP, attributable portion, SI, synergy index. RERI or AP of zero means no interaction or perfect additivity. A RERI or AP of greater than zero means positive interaction or more than additivity. A RERI or AP of less than zero means negative interaction or less than additivity. An SI of greater than one means positive interaction or more than additivity. An SI of less than one means negative interaction or less than additivity.

S22 Table

Fully adjusted models of association between functional isolation and all-cause and CVD mortality. Those with self-reported prior CVD or cancer or who died with 2 years of recruitment excluded.

| Outcome | Measure | N | Deaths (%) | HR | lci | uci |
| --- | --- | --- | --- | --- | --- | --- |
| All-cause mortality | *Functional isolation** | | | | | |
|  | No | 214,342 | 8,566 (4.0%) | 1 (ref) | - | - |
|  | Yes | 78,174 | 4,008 (5.1%) | 1.12 | 1.07 | 1.16 |
|  | | | | | | |
| CVD mortality | *Functional isolation** | | | | | |
|  | No | 329,312 | 3,140 (1.0%) | 1 (ref) | - | - |
|  | Yes | 128,834 | 1,972 (1.5%) | 1.18 | 1.05 | 1.32 |

Models adjusted for sex, ethnicity, Townsend, month of assessment, smoking, alcohol, physical activity, BMI, long-term condition count, frequency of friend and family visits, weekly group activity, and living alone. * Functional isolation defined as never able to confide in someone close or often feels lonely. HR, hazard ratio; lci, lower confidence interval; uci, upper confidence interval.

S23 Table

Fully adjusted models of association between structural component measures and all-cause and CVD mortality. Those with self-reported prior CVD or cancer or who died with 2 years of recruitment excluded.

| **Outcome** | **Measure** | **N** | **Deaths (%)** | **HR** | **lci** | **uci** |  |
| --- | --- | --- | --- | --- | --- | --- | --- |
| **All-cause mortality** | **Frequency of friends and family visits** | | | | | | |
|  | Daily | 31,417 | 1,600 (5.1%) | 1 (ref) | - | - |  |
|  | 2-4 times a week | 88,426 | 3,913 (4.4%) | 0.97 | 0.91 | 1.02 |  |
|  | Weekly | 107,893 | 4,183 (3.9%) | 0.97 | 0.92 | 1.03 |  |
|  | Monthly | 41,278 | 1,652 (4.0%) | 1.06 | 0.98 | 1.13 |  |
|  | Once every 3 months | 19,342 | 892 (4.6%) | 1.14 | 1.05 | 1.24 |  |
|  | Never | 4,160 | 334 (8.0%) | 1.43 | 1.27 | 1.61 |  |
|  | **Engages in weekly group activity** | | | | | | |
|  | Yes | 206,757 | 8,608 (4.2%) | 1 (ref) | - | - |  |
|  | No | 85,759 | 3,966 (4.6%) | 1.13 | 1.09 | 1.17 |  |
|  | **Lives alone** | | | | | | |
|  | No | 242,725 | 9,431 (3.9%) | 1 (ref) | - | - |  |
|  | Yes | 49,791 | 3,143 (6.3%) | 1.23 | 1.18 | 1.28 |  |
|  | **Structural isolation*** | | | | | | |
|  | No | 159,142 | 6,024 (3.8%) | 1 (ref) |  |  |  |
|  | Yes | 133,374 | 6,550 (4.9%) | 1.15 | 1.11 | 1.20 |  |
|  |  |  |  |  |  |  |  |
| **CVD mortality** | **Frequency of friends and family visits** | | | | | | |
|  | Daily | 31,417 | 194 (0.6%) | 1 (ref) |  |  |  |
|  | 2-4 times a week | 88,426 | 413 (0.5%) | 0.83 | 0.70 | 0.99 |  |
|  | Weekly | 107,893 | 486 (0.5%) | 0.87 | 0.74 | 1.03 |  |
|  | Monthly | 41,278 | 187 (0.5%) | 0.89 | 0.73 | 1.09 |  |
|  | Once every 3 months | 19,342 | 122 (0.6%) | 1.10 | 0.88 | 1.39 |  |
|  | Never | 4,160 | 60 (1.4%) | 1.58 | 1.17 | 2.12 |  |
|  | **Engages in weekly group activity** | | | | | | |
|  | No | 206,757 | 982 (0.5%) | 1 (ref) |  |  |  |
|  | Yes | 85,759 | 480 (0.6%) | 1.17 | 1.05 | 1.31 |  |
|  | **Lives alone** | | | | | | |
|  | No | 242,725 | 1,025 (0.4%) | 1 (ref) |  |  |  |
|  | Yes | 49,791 | 437 (0.9%) | 1.61 | 1.43 | 1.82 |  |
|  | **Structural isolation*** | | | | | | |
|  | No | 159,142 | 633 (0.4%) | 1 (ref) |  |  |  |
|  | Yes | 133,374 | 829 (0.6%) | 1.34 | 1.20 | 1.50 |  |

Models adjusted for sex, ethnicity, Townsend, month of assessment, smoking, alcohol, physical activity, BMI, long-term condition count, new dichotomous functional isolation variable – never able to confide in someone close OR often feeling lonely, and mutually for frequency of friend and family visits, weekly group activity, and living alone. HR, hazard ratio; lci, lower confidence interval; uci, upper confidence interval.

S24 Table

Fully adjusted models of joint association between frequency of friends and family visits, engaging in weekly group activity, and adverse health outcomes. Those with self-reported prior CVD or cancer or who died with 2 years of recruitment excluded.

| Outcome | Weekly group activity | Frequency of friends and family visits | HR | lci | uci |
| --- | --- | --- | --- | --- | --- |
| All-cause mortality | Yes | Daily | 1 (ref) |  |  |
|  |  | 2-4 times a week | 0.99 | 0.93 | 1.07 |
|  |  | Weekly | 1.01 | 0.94 | 1.08 |
|  |  | Monthly | 1.09 | 1.00 | 1.19 |
|  |  | Once every 3 months | 1.16 | 1.04 | 1.29 |
|  |  | Never | 1.64 | 1.40 | 1.92 |
|  | No | Daily | 1.24 | 1.11 | 1.38 |
|  |  | 2-4 times a week | 1.12 | 1.03 | 1.22 |
|  |  | Weekly | 1.12 | 1.03 | 1.21 |
|  |  | Monthly | 1.22 | 1.10 | 1.35 |
|  |  | Once every 3 months | 1.34 | 1.19 | 1.52 |
|  |  | Never | 1.46 | 1.23 | 1.74 |
|  |  |  |  |  |  |
| CVD mortality | Yes | Daily | 1 (ref) |  |  |
|  |  | 2-4 times a week | 0.82 | 0.67 | 1.00 |
|  |  | Weekly | 0.81 | 0.66 | 0.99 |
|  |  | Monthly | 0.92 | 0.72 | 1.18 |
|  |  | Once every 3 months | 1.06 | 0.80 | 1.41 |
|  |  | Never | 1.72 | 1.17 | 2.51 |
|  | No | Daily | 1.07 | 0.78 | 1.46 |
|  |  | 2-4 times a week | 0.92 | 0.72 | 1.18 |
|  |  | Weekly | 1.10 | 0.88 | 1.38 |
|  |  | Monthly | 0.91 | 0.67 | 1.23 |
|  |  | Once every 3 months | 1.28 | 0.93 | 1.78 |
|  |  | Never | 1.58 | 1.03 | 2.41 |

Models adjusted for sex, ethnicity, Townsend, month of assessment, smoking, alcohol, physical activity, BMI, long-term condition count, living alone, and functional isolation. HR, hazard ratio; lci, lower confidence interval; uci, upper confidence interval.

S25 Table

Interaction estimates for adverse health outcomes for binary exposures of frequency of friends and family visits (≥ monthly vs < monthly) and weekly group activity (yes vs no). Those with self-reported prior CVD or cancer or who died with 2 years of recruitment excluded.

| All-cause mortality | Multiplicative scale | 0.96 [0.85, 1.09] |
| --- | --- | --- |
|  | RERI | -0.02 [-0.17, 0.14] |
|  | AP | -0.01 [-0.14, 0.09] |
|  | SI | 0.95 [0.62, 1.46] |
|  | | |
| CVD mortality | Multiplicative scale | 0.96 [0.70, 1.32] |
|  | RERI | 0.01 [-0.46, 0.50] |
|  | AP | 0.00 [-0.36, 0.23] |
|  | SI | 1.01 [0.46, 2.21] |

Models adjusted for sex, ethnicity, Townsend, month of assessment, smoking, alcohol, physical activity, BMI, long-term condition count, living alone, and functional isolation. Estimates given as Hazard ratios [95% confidence intervals]). RERI, relative excess risk for interaction; AP, attributable portion, SI, synergy index. RERI or AP of zero means no interaction or perfect additivity. A RERI or AP of greater than zero means positive interaction or more than additivity. A RERI or AP of less than zero means negative interaction or less than additivity. An SI of greater than one means positive interaction or more than additivity. An SI of less than one means negative interaction or less than additivity.

S26 Table

Fully adjusted models of joint association between frequency of friends and family visits, living alone, and adverse health outcomes. Those with self-reported prior CVD or cancer or who died with 2 years of recruitment excluded.

| Outcome | Lives alone | Frequency of friends and family visits | N | Deaths (%) | HR | lci | uci |
| --- | --- | --- | --- | --- | --- | --- | --- |
| All-cause mortality | No | Daily | 24,157 | 1,098 (4.5%) | 1 (ref) |  |  |
|  |  | 2-4 times a week | 71,437 | 2,933 (4.1%) | 0.97 | 0.91 | 1.04 |
|  |  | Weekly | 92,002 | 3,250 (3.5%) | 0.95 | 0.89 | 1.02 |
|  |  | Monthly | 35,957 | 1,313 (3.7%) | 1.03 | 0.95 | 1.11 |
|  |  | Once every 3 months | 16,233 | 671 (4.1%) | 1.13 | 1.02 | 1.24 |
|  |  | Never | 2,939 | 166 (5.6%) | 1.20 | 1.02 | 1.42 |
|  | Yes | Daily | 7,260 | 502 (6.9%) | 1.17 | 1.05 | 1.30 |
|  |  | 2-4 times a week | 16,989 | 980 (5.8%) | 1.11 | 1.01 | 1.21 |
|  |  | Weekly | 15,891 | 933 (5.9%) | 1.22 | 1.11 | 1.33 |
|  |  | Monthly | 5,321 | 339 (6.4%) | 1.35 | 1.19 | 1.52 |
|  |  | Once every 3 months | 3,109 | 221 (7.1%) | 1.37 | 1.19 | 1.59 |
|  |  | Never | 1,221 | 168 (13.8%) | 2.09 | 1.77 | 2.47 |
|  |  |  |  |  |  |  |  |
| CVD mortality | No | Daily | 24,157 | 121 (0.5%) | 1 (ref) |  |  |
|  |  | 2-4 times a week | 71,437 | 297 (0.4%) | 0.86 | 0.70 | 1.07 |
|  |  | Weekly | 92,002 | 350 (0.4%) | 0.85 | 0.69 | 1.04 |
|  |  | Monthly | 35,957 | 144 (0.4%) | 0.90 | 0.71 | 1.15 |
|  |  | Once every 3 months | 16,233 | 88 (0.5%) | 1.15 | 0.87 | 1.51 |
|  |  | Never | 2,939 | 25 (0.9%) | 1.31 | 0.85 | 2.02 |
|  | Yes | Daily | 7,260 | 73 (1.0%) | 1.62 | 1.21 | 2.17 |
|  |  | 2-4 times a week | 16,989 | 116 (0.7%) | 1.22 | 0.95 | 1.58 |
|  |  | Weekly | 15,891 | 136 (0.9%) | 1.54 | 1.20 | 1.97 |
|  |  | Monthly | 5,321 | 43 (0.8%) | 1.39 | 0.98 | 1.98 |
|  |  | Once every 3 months | 3,109 | 34 (1.1%) | 1.63 | 1.11 | 2.40 |
|  |  | Never | 1,221 | 35 (2.9%) | 2.99 | 2.03 | 4.42 |

Adjusted for sex, ethnicity, Townsend, month of assessment, smoking, alcohol, physical activity, BMI, long-term condition count, weekly group activity, and functional isolation. HR, hazard ratio; lci, lower confidence interval; uci, upper confidence interval.

S27 Table

Interaction estimates for adverse health outcomes for binary exposures of friends and family visits less than monthly and living alone. Those with self-reported prior CVD or cancer or who died with 2 years of recruitment excluded.

|  |
| --- |

|  | **Interaction** | **Estimate** |
| --- | --- | --- |
| All-cause mortality | Multiplicative scale | 1.17 [1.03, 1.33] |
|  | RERI | 0.27 [0.09, 0.47] |
|  | AP | 0.16 [0.05, 0.25] |
|  | SI | 1.72 [1.20, 2.47] |
|  | | |
| CVD mortality | Multiplicative scale | 1.11 [0.80, 1.54] |
|  | RERI | 0.44 [-0.16, 1.15] |
|  | AP | 0.19 [-0.11, 0.37] |
|  | SI | 1.47 [0.87, 2.48] |

Models adjusted for sex, ethnicity, Townsend, month of assessment, smoking, alcohol, physical activity, BMI, long-term condition count, weekly group activity, and functional isolation. Estimates given as Hazard ratios [95% confidence intervals]). RERI, relative excess risk for interaction; AP, attributable portion, SI, synergy index. RERI or AP of zero means no interaction or perfect additivity. A RERI or AP of greater than zero means positive interaction or more than additivity. A RERI or AP of less than zero means negative interaction or less than additivity. An SI of greater than one means positive interaction or more than additivity. An SI of less than one means negative interaction or less than additivity.

S28 Table

Fully adjusted models of association between frequency of friends and family visits and adverse health outcomes stratified by living alone. Those with self-reported prior CVD or cancer or who died with 2 years of recruitment excluded.

| Outcome | Lives alone | Frequency of friends and family visits | HR | lci | uci |  |
| --- | --- | --- | --- | --- | --- | --- |
| All-cause mortality | No | Daily | 1 (ref) |  |  |  |
|  |  | 2-4 times a week | 0.97 | 0.91 | 1.04 |  |
|  |  | Weekly | 0.96 | 0.89 | 1.03 |  |
|  |  | Monthly | 1.04 | 0.96 | 1.13 |  |
|  |  | Once every 3 months | 1.14 | 1.04 | 1.26 |  |
|  |  | Never | 1.24 | 1.05 | 1.47 |  |
|  |  | |  | | | |
|  | Yes | Daily | 1 (ref) |  |  |  |
|  |  | 2-4 times a week | 0.94 | 0.85 | 1.05 |  |
|  |  | Weekly | 1.01 | 0.91 | 1.13 |  |
|  |  | Monthly | 1.11 | 0.97 | 1.28 |  |
|  |  | Once every 3 months | 1.12 | 0.95 | 1.31 |  |
|  |  | Never | 1.64 | 1.37 | 1.97 |  |
|  | | |  | | |  |
| CVD mortality | No | Daily | 1 (ref) |  |  |  |
|  |  | 2-4 times a week | 0.86 | 0.70 | 1.07 |  |
|  |  | Weekly | 0.85 | 0.69 | 1.05 |  |
|  |  | Monthly | 0.91 | 0.71 | 1.17 |  |
|  |  | Once every 3 months | 1.17 | 0.88 | 1.54 |  |
|  |  | Never | 1.37 | 0.89 | 2.12 |  |
|  |  | |  | | |  |
|  | Yes | Daily | 1 (ref) |  |  |  |
|  |  | 2-4 times a week | 0.76 | 0.57 | 1.02 |  |
|  |  | Weekly | 0.93 | 0.70 | 1.24 |  |
|  |  | Monthly | 0.83 | 0.57 | 1.22 |  |
|  |  | Once every 3 months | 0.96 | 0.63 | 1.45 |  |
|  |  | Never | 1.72 | 1.13 | 2.62 |  |

Models adjusted for sex, ethnicity, Townsend, month of assessment, smoking, alcohol, physical activity, BMI, long-term condition count, weekly group activity, and functional isolation. HR, hazard ratio; lci, lower confidence interval; uci, upper confidence interval.

## S29 Table

Fully adjusted models of joint association between weekly group activity, living alone, and adverse health outcomes. Those with self-reported prior CVD or cancer or who died with 2 years of recruitment excluded.

| Outcome | Lives alone | Weekly group activity | N | Deaths (%) | HR | lci | uci |
| --- | --- | --- | --- | --- | --- | --- | --- |
| All-cause mortality | No | Yes | 170,828 | 6,531 (3.8) | 1 (ref) |  |  |
|  |  | No | 71,897 | 2,900 (4.0) | 1.08 | 1.04 | 1.13 |
|  | Yes | Yes | 35,929 | 2,077 (5.8) | 1.17 | 1.11 | 1.23 |
|  |  | No | 13,862 | 1,066 (7.7) | 1.49 | 1.39 | 1.59 |
|  |  |  |  |  |  |  |  |
| CVD mortality | No | Yes | 170,828 | 700 (0.4) | 1 (ref) |  |  |
|  |  | No | 71,897 | 325 (0.5) | 1.11 | 0.97 | 1.27 |
|  | Yes | Yes | 35,929 | 282 (0.8) | 1.52 | 1.31 | 1.76 |
|  |  | No | 13,862 | 155 (1.1) | 2.03 | 1.69 | 2.44 |

Models adjusted for sex, ethnicity, Townsend, month of assessment, smoking, alcohol, physical activity, BMI, long-term condition count, frequency of friends and family visits, and functional isolation. HR, hazard ratio; lci, lower confidence interval; uci, upper confidence interval.

## S30 Table

Interaction estimates for adverse health outcomes for binary exposures of weekly group activity (yes/no) and living alone (yes/no). Those with self-reported prior CVD or cancer or who died with 2 years of recruitment excluded.

|  | **Interaction** | **Estimate** |
| --- | --- | --- |
| All-cause mortality | Multiplicative scale | 1.18 [1.08, 1.29] |
|  | RERI | 0.24 [0.13, 0.35] |
|  | AP | 0.16 [0.09, 0.23] |
|  | SI | 1.97 [1.39, 2.79] |
|  | | |
| CVD mortality | Multiplicative scale | 1.21 [0.95, 1.53] |
|  | RERI | 0.40 [0.02, 0.81] |
|  | AP | 0.20 [0.00, 0.34] |
|  | SI | 1.64 [1.00, 2.69] |

Models adjusted for sex, ethnicity, Townsend, month of assessment, smoking, alcohol, physical activity, BMI, long-term condition count, frequency of friends and family visits, and functional isolation. Estimates given as Hazard ratios [95% confidence intervals]). RERI, relative excess risk for interaction; AP, attributable portion, SI, synergy index. RERI or AP of zero means no interaction or perfect additivity. A RERI or AP of greater than zero means positive interaction or more than additivity. A RERI or AP of less than zero means negative interaction or less than additivity. An SI of greater than one means positive interaction or more than additivity. An SI of less than one means negative interaction or less than additivity.

## S31 Table

Fully adjusted models of association between weekly group activity and adverse health outcomes stratified by living alone. Those with self-reported prior CVD or cancer or who died with 2 years of recruitment excluded.

| Outcome | Living alone | Weekly group activity | N | deaths (%) | HR | lci | uci |
| --- | --- | --- | --- | --- | --- | --- | --- |
| All-cause mortality | No | Yes | 170,828 | 6,531 (3.8) | 1 (ref) |  |  |
|  |  | No | 71,897 | 2,900 (4.0) | 1.09 | 1.04 | 1.14 |
|  |  |  |  |  |  |  |  |
|  | Yes | Yes | 35,929 | 2,077 (5.8) | 1 (ref) |  |  |
|  |  | No | 13,862 | 1,066 (7.7) | 1.26 | 1.17 | 1.36 |
|  |  |  |  |  |  |  |  |
| CVD mortality | No | Yes | 170,828 | 700 (0.4) | 1 (ref) |  |  |
|  |  | No | 71,897 | 325 (0.5) | 1.12 | 0.98 | 1.28 |
|  |  |  |  |  |  |  |  |
|  | Yes | Yes | 35,929 | 282 (0.8) | 1 (ref) |  |  |
|  |  | No | 13,862 | 155 (1.1) | 1.31 | 1.07 | 1.61 |

Models adjusted for sex, ethnicity, Townsend, month of assessment, smoking, alcohol, physical activity, BMI, long-term condition count, frequency of friends and family visits, and functional isolation. HR, hazard ratio; lci, lower confidence interval; uci, upper confidence interval.

S32 Table

Fully adjusted models of joint associations between frequency of ability to confide in someone close, often feeling lonely, and structural isolation (defined as <monthly friends and family visits or not engaging in weekly group activity or living alone), and adverse health outcomes. Those with self-reported prior CVD or cancer or who died with 2 years of recruitment excluded. Models adjusted for sex, ethnicity, Townsend, month of assessment, smoking, alcohol, physical activity, BMI, long-term condition count. HR, hazard ratio; LCI, lower confidence interval; UCI, upper confidence interval.

**All-cause mortality**

| Weekly group activity, living alone, and functional isolation group | Frequency of ability to confide | N | deaths (%) | HR | lci | uci |
| --- | --- | --- | --- | --- | --- | --- |
| - not often lonely - no structural isolation | Daily | 88,438 | 3,279 (3.7%) | 1 (ref) | - | - |
|  | 2-4 times a week | 12,871 | 412 (3.2%) | 0.99 | 0.89 | 1.10 |
|  | Weekly | 12,654 | 461 (3.6%) | 1.01 | 0.92 | 1.11 |
|  | Monthly | 5,920 | 251 (4.2%) | 1.14 | 1.00 | 1.30 |
|  | Once every 3 months | 6,283 | 245 (3.9%) | 0.98 | 0.86 | 1.11 |
|  | Never | 12,613 | 644 (5.1%) | 1.09 | 1.00 | 1.19 |
| - not often lonely - structural isolation | Daily | 7,086 | 248 (3.5%) | 1.15 | 1.01 | 1.31 |
|  | 2-4 times a week | 2,466 | 84 (3.4%) | 1.28 | 1.03 | 1.59 |
|  | Weekly | 3,232 | 115 (3.6%) | 1.22 | 1.01 | 1.47 |
|  | Monthly | 1,788 | 56 (3.1%) | 1.07 | 0.82 | 1.40 |
|  | Once every 3 months | 1,594 | 50 (3.1%) | 1.00 | 0.76 | 1.33 |
|  | Never | 4,197 | 179 (4.3%) | 1.16 | 1.00 | 1.35 |
| - often lonely - no structural isolation | Daily | 55,917 | 2,406 (4.3%) | 1.15 | 1.09 | 1.21 |
|  | 2-4 times a week | 10,326 | 432 (4.2%) | 1.17 | 1.05 | 1.29 |
|  | Weekly | 11,265 | 564 (5.0%) | 1.27 | 1.16 | 1.39 |
|  | Monthly | 5,099 | 243 (4.8%) | 1.22 | 1.07 | 1.39 |
|  | Once every 3 months | 5,569 | 273 (4.9%) | 1.18 | 1.04 | 1.33 |
|  | Never | 14,337 | 977 (6.8%) | 1.41 | 1.31 | 1.51 |
| - often lonely - structural isolation | Daily | 8,432 | 393 (4.7%) | 1.27 | 1.15 | 1.42 |
|  | 2-4 times a week | 3,544 | 171 (4.8%) | 1.31 | 1.12 | 1.53 |
|  | Weekly | 5,285 | 288 (5.4%) | 1.39 | 1.23 | 1.57 |
|  | Monthly | 2,779 | 136 (4.9%) | 1.27 | 1.07 | 1.51 |
|  | Once every 3 months | 2,590 | 138 (5.3%) | 1.37 | 1.15 | 1.63 |
|  | Never | 8,231 | 529 (6.4%) | 1.48 | 1.35 | 1.62 |

**CVD mortality**

| Weekly group activity, living alone, and functional isolation group | Frequency of ability to confide | N | deaths (%) | HR | lci | uci |
| --- | --- | --- | --- | --- | --- | --- |
| - not often lonely - no structural isolation | Daily | 88,438 | 356 (0.4%) | 1.00 | - | - |
|  | 2-4 times a week | 12,871 | 38 (0.3%) | 0.91 | 0.65 | 1.27 |
|  | Weekly | 12,654 | 47 (0.4%) | 1.02 | 0.75 | 1.38 |
|  | Monthly | 5,920 | 26 (0.4%) | 1.16 | 0.78 | 1.73 |
|  | Once every 3 months | 6,283 | 19 (0.3%) | 0.72 | 0.45 | 1.14 |
|  | Never | 12,613 | 66 (0.5%) | 0.95 | 0.73 | 1.24 |
| - not often lonely - structural isolation | Daily | 7,086 | 29 (0.4%) | 1.31 | 0.90 | 1.92 |
|  | 2-4 times a week | 2,466 | 8 (0.3%) | 1.31 | 0.65 | 2.64 |
|  | Weekly | 3,232 | 13 (0.4%) | 1.45 | 0.83 | 2.53 |
|  | Monthly | 1,788 | 5 (0.3%) | 1.02 | 0.42 | 2.46 |
|  | Once every 3 months | 1,594 | 3 (0.2%) | 0.62 | 0.20 | 1.93 |
|  | Never | 4,197 | 23 (0.5%) | 1.36 | 0.89 | 2.08 |
| - often lonely - no structural isolation | Daily | 55,917 | 296 (0.5%) | 1.31 | 1.12 | 1.52 |
|  | 2-4 times a week | 10,326 | 45 (0.4%) | 1.22 | 0.89 | 1.66 |
|  | Weekly | 11,265 | 66 (0.6%) | 1.48 | 1.13 | 1.93 |
|  | Monthly | 5,099 | 22 (0.4%) | 1.06 | 0.69 | 1.64 |
|  | Once every 3 months | 5,569 | 31 (0.6%) | 1.24 | 0.86 | 1.79 |
|  | Never | 14,337 | 145 (1.0%) | 1.77 | 1.45 | 2.15 |
| - often lonely - structural isolation | Daily | 8,432 | 46 (0.5%) | 1.49 | 1.09 | 2.04 |
|  | 2-4 times a week | 3,544 | 21 (0.6%) | 1.68 | 1.08 | 2.62 |
|  | Weekly | 5,285 | 33 (0.6%) | 1.65 | 1.15 | 2.36 |
|  | Monthly | 2,779 | 17 (0.6%) | 1.60 | 0.98 | 2.62 |
|  | Once every 3 months | 2,590 | 17 (0.7%) | 1.61 | 0.99 | 2.63 |
|  | Never | 8,231 | 90 (1.1%) | 2.19 | 1.73 | 2.77 |

S33 Table

Fully adjusted models of joint associations between frequency of friends and family visits, weekly group activity, living alone, functional isolation (defined as either never able to confide in someone close or often feeling lonely), and adverse health outcomes. Those with self-reported prior CVD or cancer or who died with 2 years of recruitment excluded. Models adjusted for sex, ethnicity, Townsend, month of assessment, smoking, alcohol, physical activity, BMI, long-term condition count. HR, hazard ratio; LCI, lower confidence interval; UCI, upper confidence interval.

All-cause mortality

| Weekly group activity, living alone, and functional isolation category | Frequency of friends and family visits | N | deaths (%) | HR | LCI | UCI |
| --- | --- | --- | --- | --- | --- | --- |
| - weekly group activity - not living alone - no functional isolation | Daily | 14,161 | 618 (4.4%) | 1 (ref) |  |  |
|  | 2-4 times a week | 43,448 | 1,731 (4.0%) | 1.00 | 0.91 | 1.10 |
|  | Weekly | 50,687 | 1,699 (3.4%) | 0.96 | 0.88 | 1.06 |
|  | Monthly | 17,870 | 600 (3.4%) | 1.02 | 0.91 | 1.15 |
|  | Once every 3 months | 6,974 | 286 (4.1%) | 1.20 | 1.04 | 1.38 |
|  | Never | 717 | 47 (6.6%) | 1.59 | 1.18 | 2.13 |
| - weekly group activity - lives alone - no functional isolation | Daily | 3,558 | 205 (5.8%) | 1.08 | 0.92 | 1.27 |
|  | 2-4 times a week | 8,061 | 390 (4.8%) | 1.07 | 0.94 | 1.21 |
|  | Weekly | 6,165 | 292 (4.7%) | 1.16 | 1.01 | 1.34 |
|  | Monthly | 1,864 | 100 (5.4%) | 1.35 | 1.09 | 1.66 |
|  | Once every 3 months | 846 | 50 (5.9%) | 1.45 | 1.08 | 1.93 |
|  | Never | 158 | 21 (13.3%) | 2.38 | 1.54 | 3.67 |
| - weekly group activity - not living alone - functional isolation | Daily | 3,175 | 150 (4.7%) | 1.13 | 0.94 | 1.35 |
|  | 2-4 times a week | 9,688 | 410 (4.2%) | 1.04 | 0.92 | 1.18 |
|  | Weekly | 14,210 | 578 (4.1%) | 1.13 | 1.01 | 1.26 |
|  | Monthly | 5,903 | 238 (4.0%) | 1.19 | 1.02 | 1.38 |
|  | Once every 3 months | 3,189 | 126 (4.0%) | 1.17 | 0.97 | 1.42 |
|  | Never | 806 | 48 (6.0%) | 1.51 | 1.12 | 2.02 |
| - weekly group activity - living alone - functional isolation | Daily | 1,851 | 128 (6.9%) | 1.25 | 1.04 | 1.52 |
|  | 2-4 times a week | 4,934 | 312 (6.3%) | 1.25 | 1.09 | 1.44 |
|  | Weekly | 5,085 | 309 (6.1%) | 1.31 | 1.14 | 1.50 |
|  | Monthly | 1,703 | 121 (7.1%) | 1.55 | 1.28 | 1.89 |
|  | Once every 3 months | 1,181 | 76 (6.4%) | 1.32 | 1.04 | 1.67 |
|  | Never | 523 | 73 (14.0%) | 2.32 | 1.81 | 2.96 |
| - no weekly group activity - not living alone - no functional isolation | Daily | 5,139 | 249 (4.8%) | 1.18 | 1.01 | 1.36 |
|  | 2-4 times a week | 14,091 | 592 (4.2%) | 1.09 | 0.97 | 1.22 |
|  | Weekly | 20,215 | 683 (3.4%) | 1.02 | 0.92 | 1.14 |
|  | Monthly | 8,692 | 316 (3.6%) | 1.14 | 0.99 | 1.30 |
|  | Once every 3 months | 3,901 | 163 (4.2%) | 1.28 | 1.08 | 1.52 |
|  | Never | 616 | 34 (5.5%) | 1.25 | 0.88 | 1.76 |
| - no weekly group activity - living alone - no functional isolation | Daily | 1,079 | 93 (8.6%) | 1.59 | 1.28 | 1.98 |
|  | 2-4 times a week | 2,328 | 139 (6.0%) | 1.28 | 1.06 | 1.54 |
|  | Weekly | 2,437 | 161 (6.6%) | 1.55 | 1.30 | 1.85 |
|  | Monthly | 815 | 51 (6.3%) | 1.50 | 1.13 | 2.00 |
|  | Once every 3 months | 439 | 35 (8.0%) | 1.76 | 1.25 | 2.48 |
|  | Never | 81 | 11 (13.6%) | 2.87 | 1.58 | 5.22 |
| - no weekly group activity - not living alone - functional isolation | Daily | 1,682 | 81 (4.8%) | 1.22 | 0.97 | 1.54 |
|  | 2-4 times a week | 4,210 | 200 (4.8%) | 1.19 | 1.02 | 1.40 |
|  | Weekly | 6,890 | 290 (4.2%) | 1.20 | 1.04 | 1.37 |
|  | Monthly | 3,492 | 159 (4.6%) | 1.37 | 1.15 | 1.63 |
|  | Once every 3 months | 2,169 | 96 (4.4%) | 1.36 | 1.09 | 1.68 |
|  | Never | 800 | 37 (4.6%) | 1.14 | 0.82 | 1.59 |
| - no weekly group activity - living alone - functional isolation | Daily | 772 | 76 (9.8%) | 1.74 | 1.37 | 2.20 |
|  | 2-4 times a week | 1,666 | 139 (8.3%) | 1.54 | 1.28 | 1.85 |
|  | Weekly | 2,204 | 171 (7.8%) | 1.65 | 1.39 | 1.96 |
|  | Monthly | 939 | 67 (7.1%) | 1.68 | 1.30 | 2.16 |
|  | Once every 3 months | 643 | 60 (9.3%) | 1.97 | 1.51 | 2.57 |
|  | Never | 459 | 63 (13.7%) | 2.53 | 1.95 | 3.28 |

CVD mortality

| Weekly group activity, living alone, and functional isolation category | Frequency of F&FVs | N | deaths (%) | HR | LCI | UCI |
| --- | --- | --- | --- | --- | --- | --- |
| - weekly group activity - not living alone - no functional isolation | Daily | 14,161 | 71 (0.5%) | 1.00 |  |  |
|  | 2-4 times a week | 43,448 | 173 (0.4%) | 0.84 | 0.64 | 1.11 |
|  | Weekly | 50,687 | 175 (0.3%) | 0.79 | 0.60 | 1.05 |
|  | Monthly | 17,870 | 67 (0.4%) | 0.89 | 0.64 | 1.24 |
|  | Once every 3 months | 6,974 | 34 (0.5%) | 1.08 | 0.71 | 1.62 |
|  | Never | 717 | 5 (0.7%) | 1.20 | 0.49 | 2.99 |
| - weekly group activity - lives alone - no functional isolation | Daily | 3,558 | 35 (1.0%) | 1.74 | 1.16 | 2.62 |
|  | 2-4 times a week | 8,061 | 42 (0.5%) | 1.06 | 0.73 | 1.56 |
|  | Weekly | 6,165 | 31 (0.5%) | 1.07 | 0.70 | 1.64 |
|  | Monthly | 1,864 | 12 (0.6%) | 1.34 | 0.73 | 2.48 |
|  | Once every 3 months | 846 | 7 (0.8%) | 1.59 | 0.73 | 3.47 |
|  | Never | 158 | 5 (3.2%) | 4.18 | 1.68 | 10.37 |
| - weekly group activity - not living alone - functional isolation | Daily | 3,175 | 19 (0.6%) | 1.18 | 0.71 | 1.96 |
|  | 2-4 times a week | 9,688 | 44 (0.5%) | 0.93 | 0.64 | 1.35 |
|  | Weekly | 14,210 | 59 (0.4%) | 0.91 | 0.64 | 1.28 |
|  | Monthly | 5,903 | 25 (0.4%) | 0.98 | 0.62 | 1.54 |
|  | Once every 3 months | 3,189 | 20 (0.6%) | 1.42 | 0.86 | 2.33 |
|  | Never | 806 | 8 (1.0%) | 1.82 | 0.88 | 3.79 |
| - weekly group activity - living alone - functional isolation | Daily | 1,851 | 15 (0.8%) | 1.29 | 0.74 | 2.26 |
|  | 2-4 times a week | 4,934 | 42 (0.9%) | 1.47 | 1.00 | 2.15 |
|  | Weekly | 5,085 | 46 (0.9%) | 1.61 | 1.11 | 2.33 |
|  | Monthly | 1,703 | 20 (1.2%) | 2.00 | 1.21 | 3.29 |
|  | Once every 3 months | 1,181 | 11 (0.9%) | 1.41 | 0.75 | 2.67 |
|  | Never | 523 | 16 (3.1%) | 3.32 | 1.92 | 5.75 |
| - no weekly group activity - not living alone - no functional isolation | Daily | 5,139 | 19 (0.4%) | 0.80 | 0.48 | 1.32 |
|  | 2-4 times a week | 14,091 | 62 (0.4%) | 0.96 | 0.68 | 1.35 |
|  | Weekly | 20,215 | 82 (0.4%) | 0.96 | 0.70 | 1.33 |
|  | Monthly | 8,692 | 31 (0.4%) | 0.84 | 0.55 | 1.28 |
|  | Once every 3 months | 3,901 | 21 (0.5%) | 1.21 | 0.74 | 1.97 |
|  | Never | 616 | 9 (1.5%) | 2.27 | 1.13 | 4.56 |
| - no weekly group activity - living alone - no functional isolation | Daily | 1,079 | 13 (1.2%) | 2.12 | 1.17 | 3.83 |
|  | 2-4 times a week | 2,328 | 17 (0.7%) | 1.46 | 0.86 | 2.49 |
|  | Weekly | 2,437 | 25 (1.0%) | 2.05 | 1.30 | 3.24 |
|  | Monthly | 815 | 4 (0.5%) | 0.96 | 0.35 | 2.63 |
|  | Once every 3 months | 439 | 6 (1.4%) | 2.32 | 1.01 | 5.36 |
|  | Never | 81 | 0 | - | - | - |
| - no weekly group activity - not living alone - functional isolation | Daily | 1,682 | 12 (0.7%) | 1.57 | 0.85 | 2.90 |
|  | 2-4 times a week | 4,210 | 18 (0.4%) | 0.92 | 0.55 | 1.55 |
|  | Weekly | 6,890 | 34 (0.5%) | 1.12 | 0.74 | 1.68 |
|  | Monthly | 3,492 | 21 (0.6%) | 1.39 | 0.85 | 2.27 |
|  | Once every 3 months | 2,169 | 13 (0.6%) | 1.39 | 0.77 | 2.52 |
|  | Never | 800 | 3 (0.4%) | 0.66 | 0.21 | 2.09 |
| - no weekly group activity - living alone - functional isolation | Daily | 772 | 10 (1.3%) | 1.99 | 1.02 | 3.87 |
|  | 2-4 times a week | 1,666 | 15 (0.9%) | 1.50 | 0.86 | 2.63 |
|  | Weekly | 2,204 | 34 (1.5%) | 2.73 | 1.81 | 4.12 |
|  | Monthly | 939 | 7 (0.7%) | 1.35 | 0.62 | 2.93 |
|  | Once every 3 months | 643 | 10 (1.6%) | 2.47 | 1.27 | 4.81 |
|  | Never | 459 | 14 (3.1%) | 3.91 | 2.19 | 6.98 |

S34 Table

Fully adjusted models of joint association between functional and structural isolation and adverse health outcomes. Those with self-reported prior CVD or cancer or who died with 2 years of recruitment excluded.

| Outcome | Functional isolation | Structural isolation | N | Deaths (%) | HR | lci | uci |
| --- | --- | --- | --- | --- | --- | --- | --- |
| All-cause mortality | No | No | 126,166 | 4,648 (3.7%) | 1 (ref) | - | - |
|  | Yes | No | 32,976 | 1,376 (4.2%) | 1.12 | 1.05 | 1.19 |
|  | No | Yes | 88,176 | 3,918 (4.4%) | 1.17 | 1.12 | 1.22 |
|  | Yes | Yes | 45,198 | 2,632 (5.8%) | 1.37 | 1.31 | 1.44 |
|  |  |  |  |  |  |  |  |
| CVD mortality | No | No | 126,166 | 486 (0.4%) | 1 (ref) | - | - |
|  | Yes | No | 32,976 | 147 (0.4%) | 1.12 | 0.93 | 1.34 |
|  | No | Yes | 88,176 | 460 (0.5%) | 1.32 | 1.16 | 1.50 |
|  | Yes | Yes | 45,198 | 369 (0.8%) | 1.79 | 1.56 | 2.06 |

Models adjusted for sex, ethnicity, Townsend, month of assessment, smoking, alcohol, physical activity, BMI, long-term condition count. HR, hazard ratio; lci, lower confidence interval; uci, upper confidence interval.

S35 Table

Interaction estimates for adverse health outcomes for binary exposures of functional and structural isolation. Those with self-reported prior CVD or cancer or who died with 2 years of recruitment excluded.

|  | **Interaction** | **Estimate** |
| --- | --- | --- |
| All-cause mortality | Multiplicative scale | 1.05 [0.97, 1.14] |
|  | RERI | 0.09 [-0.01, 0.18] |
|  | AP | 0.06 [-0.01, 0.13] |
|  | SI | 1.30 [0.96, 1.77] |
|  | | |
| CVD mortality | Multiplicative scale | 1.22 [0.97, 1.54] |
|  | RERI | 0.36 [0.06, 0.65] |
|  | AP | 0.20 [0.03, 0.34] |
|  | SI | 1.84 [0.98, 3.45] |

Models adjusted for sex, ethnicity, Townsend, month of assessment, smoking, alcohol, physical activity, BMI, long-term condition count. Estimates given as Hazard ratios [95% confidence intervals]). RERI, relative excess risk for interaction; AP, attributable portion, SI, synergy index. RERI or AP of zero means no interaction or perfect additivity. A RERI or AP of greater than zero means positive interaction or more than additivity. A RERI or AP of less than zero means negative interaction or less than additivity. An SI of greater than one means positive interaction or more than additivity. An SI of less than one means negative interaction or less than additivity.
